# Machine Learning for the Analysis of Healthy Lifestyle Data: Scoping Review and Guidelines

**DOI:** 10.2196/78648

**Published:** 2026-02-27

**Authors:** Tony Estrella, Lluis Capdevila, Carla Alfonso, Josep-Maria Losilla

**Affiliations:** 1 Sport Research Institute, Universitat Autònoma de Barcelona, Bellaterra, Spain; 2 Department of Basic Psychology, Universitat Autònoma de Barcelona, Edifici N, Planta 1, Cerdanyola del Vallès, Barcelona, 08193, Spain, 34 93 581 2758; 3 Department of Psychobiology and Methodology of Health Science, Universitat Autònoma de Barcelona, Cerdanyola del Vallès, Barcelona, Spain

**Keywords:** machine learning, artificial intelligence, healthy lifestyle, physical activity, diet, sleep, stress, review, data analysis, XAI, explainable artificial intelligence

## Abstract

**Background:**

Advances in data science and technology have transformed lifestyle research by enabling the integration of multimodal information and the generation of large-scale datasets. Despite the growing interest in machine learning (ML) within health behavior research, significant methodological gaps remain.

**Objective:**

The study aims to systematically review the applications of supervised ML algorithms in the analysis of healthy lifestyle data, with a particular focus on the methodological approaches used. The specific objectives are to explore the types and sources of data used for health outcomes, examine the ML processes used, including explainable artificial intelligence (XAI) methods, and review the software tools used. Additionally, this review aims to provide practical guidelines to enhance the quality and transparency of future ML research in health.

**Methods:**

Following the PRISMA-ScR (Preferred Reporting Items for Systematic Reviews and Meta-Analyses extension for Scoping Reviews) recommendations, the search was conducted across PubMed, PsycINFO, and Web of Science, yielding 65 studies that met the inclusion criteria.

**Results:**

Most studies (48/65, 74%) integrated multidomain data from physical activity, diet, sleep, and stress. Data sources were split between self-acquired data (33/65, 51%) and health repositories (32/65, 49%). Single-item measurements were common, particularly for physical activity, diet, and sleep. Although 40 of 65 studies used a multimodel approach, random forest was the most frequently applied algorithm. To improve explainability, 22 of 65 (33.84%) studies incorporated specific XAI methods, with 21 using Shapley Additive Explanation values and 1 using local interpretable model-agnostic explanations. R (R Core Team) and Python (Python Software Foundation) were the most widely used software tools, with variation in the libraries used.

**Conclusions:**

This review highlights methodological gaps in the application of supervised ML to healthy lifestyle data. The ML workflow should span from data acquisition to explainability, using iterative steps to improve methodological rigor. Although multidomain data collection enhances the understanding of health issues related to lifestyle, representativeness remains limited due to methodological shortcomings in data acquisition. While random forest was the most commonly used algorithm, a multimodel approach is recommended for a comprehensive comparison. Lifestyle components consistently ranked among the top features in studies integrating XAI. Incorporating XAI methods into the ML pipeline can support personalized interventions, provided data collection is accurate. The R metapackage (tidymodels; Max Kuhn and Hadley Wickham) facilitates process evaluation through unified syntax, improving replicability. Methodological and reporting guidelines and a checklist are provided to enhance transparency and replicability in multidisciplinary ML research.

## Introduction

There is a growing interest in understanding the effects of synergistic relationships among lifestyle behaviors and their effect on health outcomes [1,2]. Traditionally, healthy lifestyle (HL) research has primarily focused on physical activity and diet. However, recent studies increasingly include sleep and stress management as critical components of lifestyle [3,4]. For instance, stress has been shown to negatively influence physical activity, sleep, and dietary habits [5], which in turn have an overall impact on health and well-being. This multidimensional perspective has gained attention in public health under the concept of lifestyle medicine, which incorporates physical activity, diet, sleep, and stress management as cost-effective interventions to prevent noncommunicable diseases, such as cardiovascular and metabolic diseases [6-8].

Technological advances, including wearable devices and lifelogging processes, have significantly enhanced the capability to collect multimodal, high-frequency, and ecological lifestyle data [9,10]. This wealth of data provides valuable contextual information and insights for researchers and users [11]. However, the vast amount and complexity of behavioral and physiological data expose significant analytical challenges. Traditional statistical models often struggle with the high dimensionality, heterogeneity, and nonlinearity typical of lifestyle studies. Recent progress in computational power and artificial intelligence (AI), particularly machine learning (ML), has contributed to addressing these limitations [12].

ML models are capable of analyzing complex data types and generating insights and knowledge to improve decision-making [13,14]. Furthermore, ML algorithms can flexibly handle nonlinear relationships among features and outcomes. While the boundary between classical statistics and ML is not clear, ML algorithms are recognized for their flexible data-driven approach, avoiding the imposition of a predetermined relational structure between variables [15-17]. Additionally, prioritizing algorithms that maximize generalizability to new data, often referred to as scalability in the big data context, is crucial to face new health challenges [18,19]. These characteristics make ML analysis a suitable methodology for predictive modeling and feature extraction in health-related lifestyle research.

ML models are broadly classified into supervised learning (SL) and unsupervised learning (UL). In SL, the model is trained with labeled data, where each observation has an associated response measurement, to predict known outcomes such as disease risk or behavioral adherence [19]. The goal of SL is to fit a model that can predict the response when applied to new data. When the response value is continuous, this is known as a “regression problem”; when the response is categorical, it is known as a “classification problem.” In contrast, in UL models, the goal is to discover patterns rather than predict outcomes, since there is no associated response to the input, and the model seeks relationships and similarities between observations. In the health domain, where diagnosis and detection are key focuses, SL, and particularly classification tasks, are more prevalent due to their ability to evaluate these predictions [20,21]. Clinical applications of SL include triage systems, prognosis prediction, and disease classification using rapid testing [22]. Consequently, SL methods are standard in epidemiology to enhance clinical decisions based on input-output relationships [23]. Since prediction and explainability are central concerns in health research, this scoping review focuses specifically on SL methods.

Despite the growing attention to ML in health behavior research, there remain significant methodological gaps. Prior reviews have focused primarily on outcome effectiveness or AI chatbot interventions, often providing limited detail about the ML process involved [24]. A recent scoping review on ML methods used in health promotion and behavioral change found that the main interventions studied are those related to physical activity, while other crucial aspects of HL were overlooked, revealing an imbalance in this literature [25]. Similarly, Lai et al [26] reviewed the applications of large language models in exercise recommendations and physical activity, highlighting methodological limitations associated with these AI models. In sum, these studies underscore the need for a more comprehensive review to include a holistic concept of HL. Furthermore, methodological details such as data preprocessing, model evaluation, and explainability are often underreported, hindering transparency, reproducibility, and interdisciplinary collaboration.

To address the lack of explainability, explainable artificial intelligence (XAI) has emerged, which focuses on understanding AI algorithms and making them more transparent. XAI aims to provide human-understandable explanations for the decisions made by ML models [27]. In HL research, XAI can be directed to identify the set of behaviors that significantly influence health, thereby enhancing transparency and trust in AI. It is important to distinguish between interpretability and explainability in the AI context. While interpretability refers to understanding the influence of each feature in the original model, explainability involves deriving actionable human insights from the model’s predictions [28]. Interpretability enables AI developers to delve into the model’s decision-making to comprehend how algorithms reach their decisions. Conversely, explainability refers to the process for creating common meaning from model decisions and therefore provides human-readable explanations [28]. Therefore, reporting the explainability method used in ML projects is crucial not only to enhance the decision-making process of the end user but also to understand how lifestyle factors interact with health outcomes.

Therefore, this study aims to systematically review the applications of supervised ML algorithms in analyzing HL data, with a specific focus on the methodological aspects used in these studies, rather than their results. The specific objectives are to explore (1) the specific lifestyle data used in health outcome studies; (2) the sources and types of data subjected to analysis; (3) the characteristics of the ML models, including XAI methods; and (4) the programs and libraries used for ML implementation. Additionally, based on the findings of this scoping review, we aim to provide practical guidelines to enhance the quality and transparency of future ML research in lifestyle science. A scoping review is the type of systematized review (ie, systematic, transparent, and replicable) most appropriate for addressing these objectives [29].

## Method

### Overview

To maximize the reporting quality of this scoping review, we followed the PRISMA-ScR (Preferred Reporting Items for Systematic Reviews and Meta-Analyses extension for Scoping Reviews) recommendations [30] (checklist provided in Checklist 1). The protocol for this scoping review was registered with the International Platform of Registered Systematic Review and Meta-Analysis Protocols (INPLASY) [31]. All data generated in this review are provided in Multimedia Appendix 1 and are accessible in the institutional repository [32].

### Search Strategy

In this scoping review, we searched for primary studies in the 3 principal health databases: PubMed (National Center for Biotechnology Information), PsycINFO (ProQuest), and Web of Science (Clarivate). The search was restricted to medical and psychological databases to capture studies directly relevant to health outcomes. Consequently, studies primarily published in engineering or computer science, which may focus on algorithm development or sensor-based data processing, were not included. The search strategy followed the PRESS (Peer Review of Electronic Search Strategies) [33] and PRISMA-S (Preferred Reporting Items for Systematic Reviews and Meta-Analyses–Search extension) guidelines [34] and consisted of 2 groups of search terms referring to (1) HL and (2) ML. We also added a third group of terms preceded by the Boolean operator “NOT” to improve the specificity of the search strategy.

This scoping review adopts a health-focused perspective, in which HL is treated as a multidimensional construct rather than the sum of isolated behaviors [6,35,36]. Therefore, the umbrella term HL was combined using the operator “OR” with an interaction block including (1) physical activity, (2) diet, (3) sleep, and (4) stress. This block is aligned with the multiple health behavior change and lifestyle medicine frameworks, in which the interaction between behaviors is a central construct [37,38].

The search strategy was adapted to the specific syntax of each database (Table S1 in Multimedia Appendix 1). The search was conducted on October 10, 2025, with language restrictions (English and Spanish) but without limitations on publication years.

### Study Selection

#### Inclusion and Exclusion Criteria

Studies were included in or excluded from the review according to the following criteria provided in Textbox 1.

Textbox 1. **Inclusion and exclusion criteria.**

**Inclusion criteria:**

- Used supervised machine learning (ML) models for analyzing lifestyle data.
- Analyzed lifestyle behaviors as either inputs or outputs of the ML models.
- Used data from real individuals (not simulations).
- Published in English or Spanish.

**Exclusion criteria:**

- Focused on unsupervised learning (UL) without connection to supervised learning (SL) modeling.
- Focused on mathematical formulation or guidelines for implementing ML models in health.
- Used simulated data or aimed to develop a chatbot or app based on ML.
- Primarily addressed substance abuse, such as alcohol intake or smoking cessation.
- Focused exclusively on classical statistical regression algorithms, such as linear or logistic regression, which were not considered ML on their own in this review.

#### Justification of Exclusions

UL algorithms were excluded because they do not have an associated response to inputs, thereby lacking performance evaluation. Classical statistical regression algorithms, such as linear or logistic regression, were not considered in this review. While the boundary between classical statistics and ML is not clear, ML algorithms are recognized for their flexible data-driven approach, avoiding the imposition of a predetermined relational structure between variables [15-17]. Additionally, prioritizing algorithms that maximize generalizability to new data, often referred to as scalability in the big data context, is crucial to address new health challenges [18,19]. Consequently, studies focusing exclusively on this type of statistical algorithm were excluded. However, we acknowledge that the use of a model ensemble approach allows for the inclusion of these statistical algorithms to assess the performance of different algorithms during the evaluation step. Studies on substance abuse disorders were excluded as they involve distinct behavioral and neurobiological mechanisms that differ substantially from the domains of physical activity, diet, sleep, and stress, which are the core components of HL behaviors as defined in this review. In addition, substance abuse is categorized within the risk avoidance cluster, which is conceptually distinct from the other 4 behaviors examined in this review [3]. This distinction is well established in multiple health behavior theories, which differentiate behaviors that enhance health from those that reduce risk through avoidance. Therefore, its exclusion preserves the applicability of results to a multiple health behavior framework, as the selected behaviors are interrelated through shared psychological resources [2,39].

Two reviewers (TE and CA) independently screened titles and abstracts in the first phase and full texts in the second phase. Discrepancies were resolved by consensus, with the participation of a third reviewer (JML) when necessary. Agreement between reviewers during the selection process was analyzed by calculating Cohen κ.

### Data Management

Mendeley was used as reference management software; the results of the search strategy were entered, and duplicates were merged or removed. An ad hoc checklist was used to extract information from the included papers. The checklist was divided into 5 sections:

- General information: authors, title, year, and country of affiliation.
- Methodological data: type of study, aim, year of data collection, form of data acquisition, sample, and countries represented in the data.
- Study variables: health issues, lifestyle features, and the model’s input and output variables.
- Software: statistical programming language, libraries, and packages.
- Model aspects: type of problem, stages of ML analysis, ML methods, model evaluation, evaluation metrics, and XAI methods.

### Strategy of Data Synthesis

The review was presented as a narrative synthesis, and the information was summarized in tables and figures. The information extracted from the studies was divided into 3 blocks: type of data, ML process, and software. For data extraction, we focused on lifestyle components, health outcomes, data sources, acquisition methods, and data typology. Regarding the ML process, we focused on the whole process, consisting of preprocessing, modeling, validation, evaluation, and XAI methods. For model evaluation, only the procedures used for final performance assessment were extracted. When studies reported cross-validation, we classified it as the final performance estimation method unless authors explicitly stated its use for hyperparameter optimization. To identify the top-ranked lifestyle components in the XAI analysis, we systematically examined the figures and tables reported in each study. In this review, the term ranked refers to features that were highlighted by the XAI algorithm. In contrast, unranked indicates that appeared in the model but were not reported in the XAI visualization, while not available denotes features that were not included in the ML model. Software used in each study was also recorded.

## Results

### Overview

**Figure 1.**
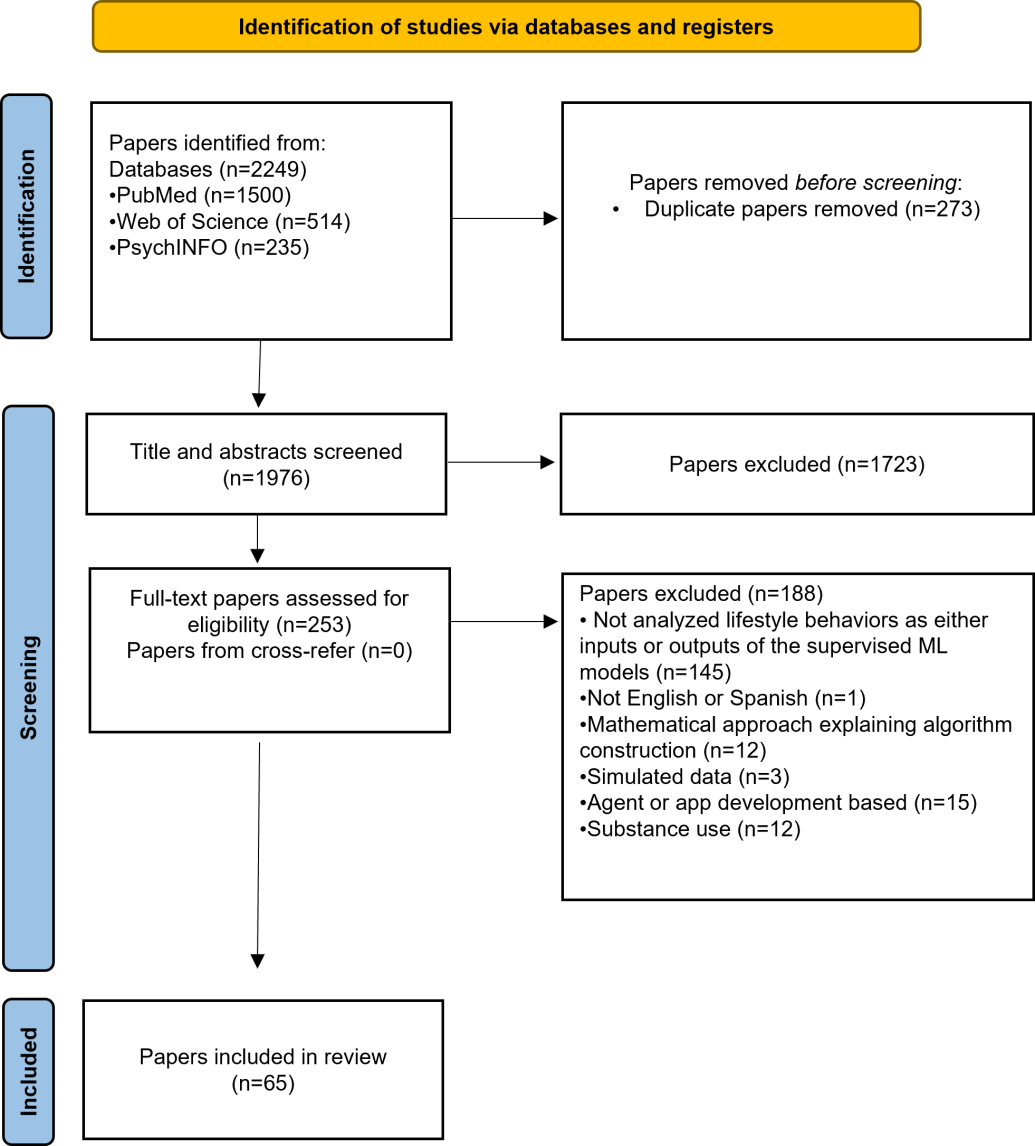
PRISMA (Preferred Reporting Items for Systematic Reviews and Meta-Analyses) flow diagram of the scientific literature search and selection. ML: machine learning.

A total of 2249 papers were retrieved from the databases, and 65 studies met the eligibility criteria and were included in this scoping review (refer to Figure 1). There was very good agreement between reviewers during the selection process: 96% (n=52; κ=0.84, 95% CI 0.61-1.0) in the title and abstract screening and 94% (n=35; κ=0.88, 95% CI 0.71-1.0) in the full-text screening.

**Figure 2.**
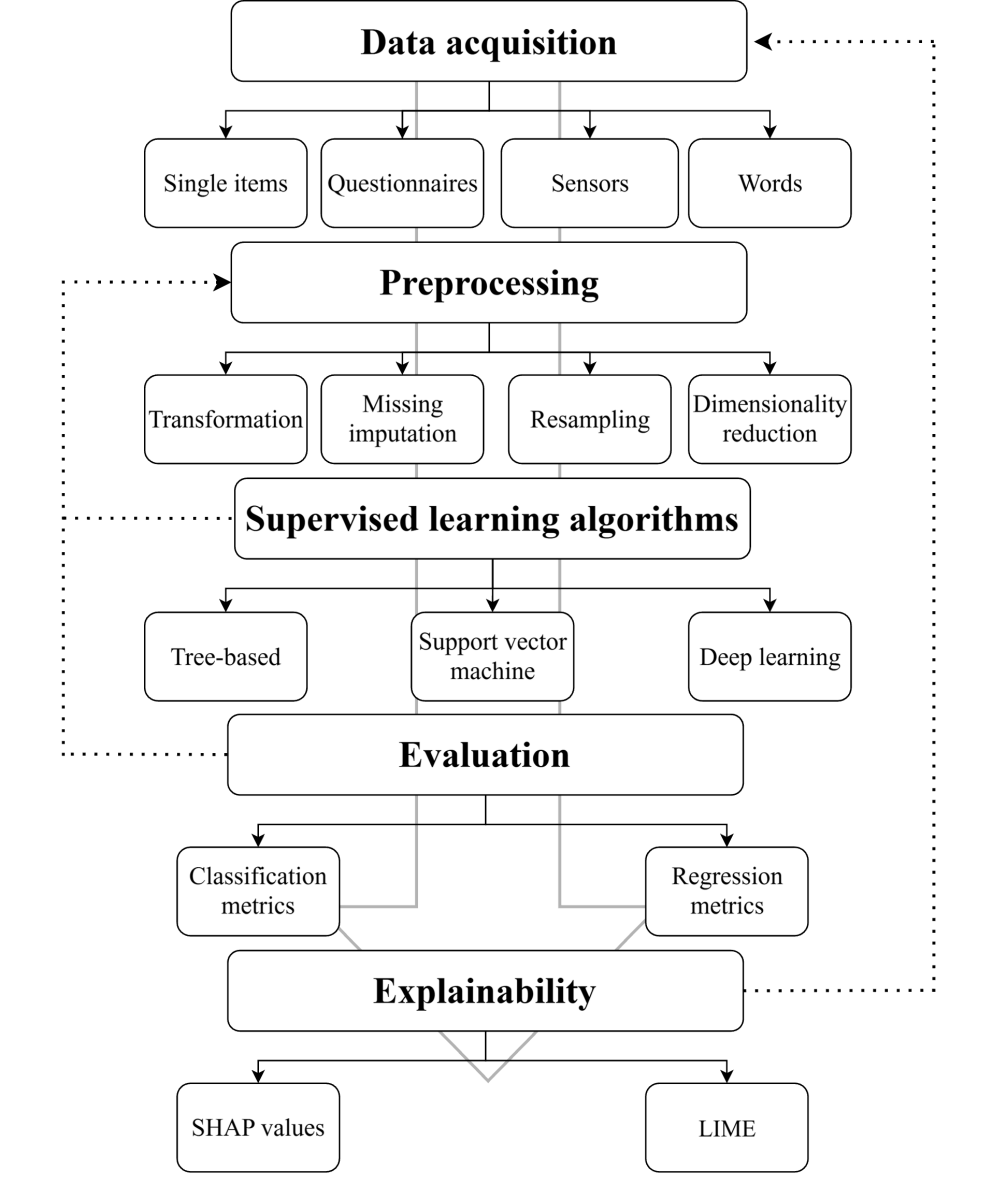
Overview of the machine learning workflow, spanning from data acquisition to explainability. The dashed lines represent iterative feedback loops within the process. LIME: local interpretable model-agnostic explanations; SHAP: Shapley Additive Explanation.

From this point forward, the “Results” section is structured following the ML workflow depicted in Figure 2, which illustrates the 5 key steps in the ML pipeline. The process begins with data acquisition, followed by preprocessing to prepare the data. Then, SL algorithms are applied and evaluated to determine their effectiveness. Finally, explainability techniques are used to understand the models. The dashed lines indicate that modeling, evaluation, and explainability can improve earlier stages, making the process iterative. Each stage of the process corresponds to a subsection. Finally, we examined the software used throughout the entire process in the included studies.

### Data Acquisition: Collection Modes, Data Typology, Lifestyle Variables, and Health Outcomes

**Figure 3.**
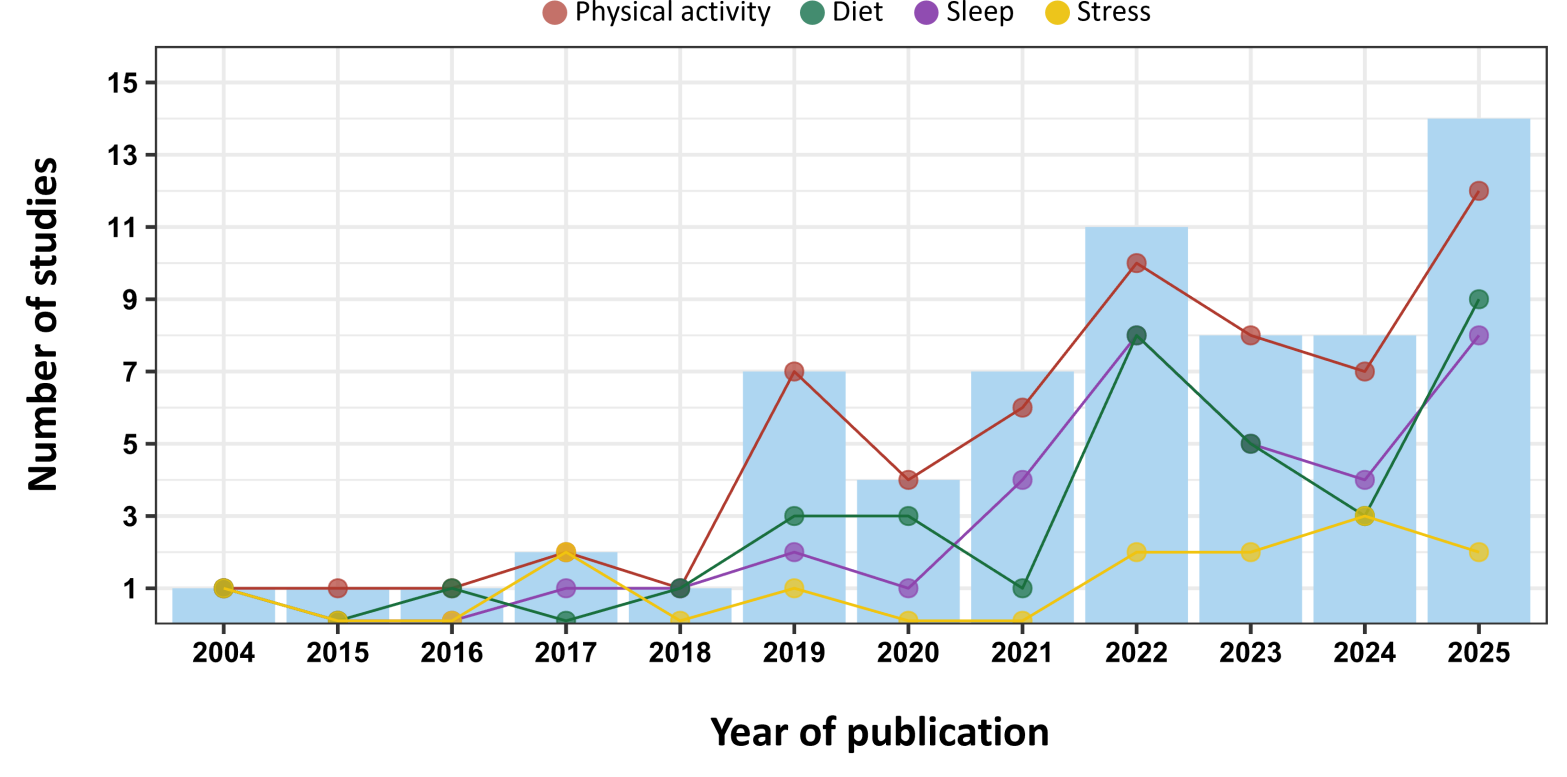
Bar graph showing productivity by publication year, with each line and dot representing a lifestyle component.

The 65 papers included in this review were published between 2004 and 2025, with 57 (87.7%) published since 2019. Figure 3 shows the annual productivity output stratified by lifestyle components. The studies were carried out in several geographical regions across 4 continents (Table S2 and Figure S1 in Multimedia Appendix 1). The mean sample size was 29,905.14 participants, with the smallest study including 8 participants and the largest including 470,778 participants.

Four lifestyle domains were identified in the review: physical activity, diet, sleep, and stress. Most studies (48/65, 74%) integrated data from more than 1 lifestyle domain (refer to Table 1). The most studied component was physical activity, featured in 61 of 65 studies. Diet and sleep appeared in 33 of 65 (51%) and 34 of 65 (52%) studies, respectively, while stress appeared in 15 of 65 (23%) studies (refer to Figure 3). Only 17 of 65 (26%) studies focused exclusively on a single domain (15 on physical activity [40-54] and 2 on sleep [55,56]).

Regarding data sources, 33 of 65 (51%) studies relied on self-acquired data, while 32 of 65 (49%) studies used either private or public health datasets, such as UK Biobank [57]. Among the studies using self-acquired data, the mean sample size was 10,503.41 participants. Six studies focused exclusively on females [46,47,58-61], and 27 studies reported a female proportion ranging from 41% to 89.4%. Studies using health repositories exhibited a greater mean sample size of 80,406.38 participants. Nineteen studies reported a female proportion ranging from 41.33% to 70%, 12 did not report sex distribution, and 1 study focused exclusively on females.

**Figure 4.**
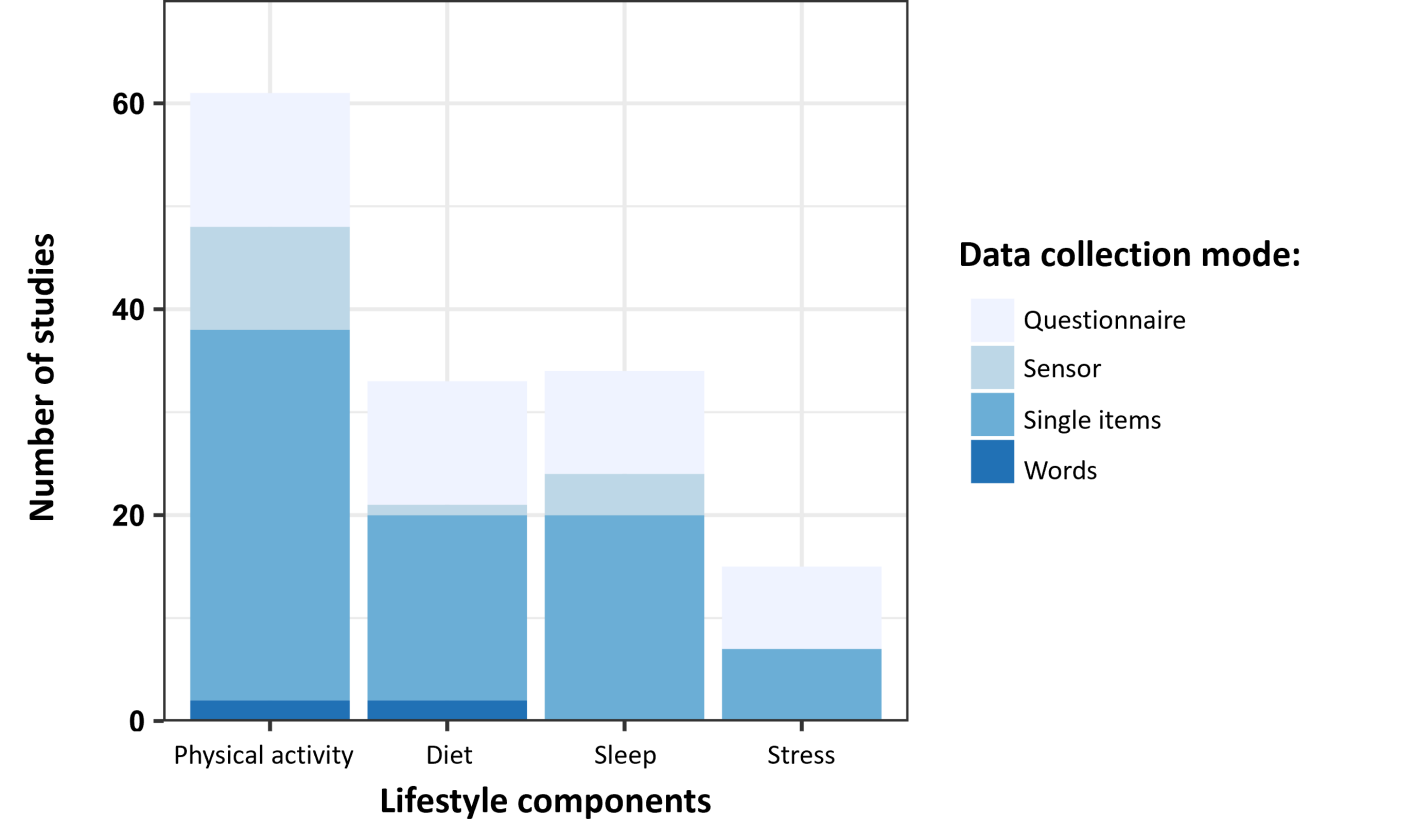
Stacked bar chart summarizing the data acquisition methodology for each lifestyle component.

Figure 4 summarizes the different data collection modes used for each of the 4 lifestyle domains identified (Table S3 in Multimedia Appendix 1 provides a detailed description of each measure). Single-item measures were used in 40 of 65 (61.5%) studies assessing physical activity, 18 of 65 (27.7%) studies assessing diet, and 22 of 65 (33.8%) studies assessing sleep. Despite the heterogeneity of these items, distinct categories emerged within each lifestyle domain.

For physical activity, the emerging categories included engagement in physical activities [47,50-52,54,58,62-66], intensity [10,42,49,57,67-71], frequency [43,44,60,72-86], and environmental factors [87,88]. Within the diet domain, categories included frequency of consumption [58,60,66,72,84], types of products [75,76,80,82], environmental factors [87], and consumption habits [59,62,65,68,70,71,86,89]. Regarding sleep, categories included sleep duration [57,60,62,66-68,70,72,77-79,81,82,84,85,87], perceived sleep quality [63,89], and sleep-related problems [69,80]. Finally, within the stress domain, the emerging category focused primarily on stress level [10,57,79,82,86,89,90].

Questionnaires were used to collect physical activity data in 13 studies. The standardized questionnaires included the Global Physical Activity Questionnaire (GPAQ) [53,72,91], the International Physical Activity Questionnaire (IPAQ) [92], the Lifetime Total Physical Activity Questionnaire (exercise and sport subscale) [93], the Nutritional and Social Healthy Habits (NutSo-HH) scale [94], the Indian Migration Study Physical Activity Questionnaire (IMS-PAQ) [95], physical fitness test [41], the Short Questionnaire to Assess Health-Enhancing Physical Activity (SQUASH) [73,74], the physical activity scale from the Active Living Index [88], the Pregnancy Physical Activity Questionnaire [61], and the Physical Activity Scale for the Elderly [96]. For diet assessment, 12 studies used the Food Frequency Questionnaire (FFQ) [57,73,74,83,88,91,92,95], the PrimeScreen questionnaire [61], the Mini Nutritional Assessment [97], the NutSo-HH scale [94], and a nonstandardized questionnaire consisting of items from different questionnaires [93]. The standardized questionnaires used to measure sleep were the Pittsburgh Sleep Quality Index [10,56,61,86,91,92,96,97], the NutSo-HH scale [94], the Munich Chronotype questionnaire and Sleep Disturbance Scale for Children [64], and the Epworth Sleepiness Scale [96]. To measure stress, the stress subscale of the Depression Anxiety Stress Scale (DASS) [92,93], the INTERHEART stress questionnaire [59], the Psychosocial Well-being Index-Short Form [91], the Perceived Stress Scale [61,64,88], and the Profile of Mood States [81] were used. Regarding data collection through sensors, most studies used wearable devices. One study used a smartphone to obtain points of interest related to physical activity and diet [98], and 1 sleep study used polysomnography [55]. Finally, 2 studies used words related to physical activity and diet, 1 derived from Google Trends [99] and the other from Twitter (Twitter, Inc) [100]*.*

Concerning the modeled inputs, 56 of 65 (86.1%) studies used multimodal data. The input modalities were lifestyle (60/65, 92.3%), sociodemographic (49/65, 75.3%), clinical (29/65, 44.6%), anthropometric (14/65, 21.5%), psychological (20/65, 30.7%), physical (3/65, 4.62%), environmental (10/65, 15.38%), physiological (3/65, 4.62%), and behavioral (2/65, 3.07%). The model outcomes included lifestyle domains in 14 (22% studies; 5 physical activity [40,44-46,90], 5 sleep [10,48,86,97,101], 3 diet [62,66,94], and 1 stress [61]) and other health outcomes in 51 (75% studies; with mental health, cancer, cardiovascular diseases, and diabetes being the most frequent categories; refer to Table 1).

Cross-sectional data were acquired in 36 (55%) studies [42-44,51-53,56-58,60-62,65,66,71-74,77-80,84,85,89,92,94,95,97], longitudinal data in 18 (28%) [10,41,50,54,55,59,64,67-69,81,82,96] studies, time-series data in 7 (11%) [45,46,48,75,76,101,102] studies, combined longitudinal and time-series data in 1 study [90], textual data in 2 studies [99,100], and combined cross-sectional and geographical data in 1 study [103].

**Table 1.** Summary of the machine learning (ML) workflow from data acquisition to explainability in the included studies.

Study	Physical activity	Diet	Sleep	Stress	Health outcome	Preprocess	ML^a^ algorithm	Model evaluation	XAI^b^
Abdul Rahman et al [72]	Questionnaire (standardized) and single items (frequency)	Single items (frequency)	Single items (sleep hours)	—	Mental health	Missing imputation, Resampling, and dimensionality reduction	RF^c^, ANN^d^, NB^e^, and KNN^f^	Hold-out test set	—
Afrash et al [58]	Single items (engagement)	Single items (frequency)	—	—	Cancer	Transformation, missing imputation, and dimensionality reduction	DT^g^, MLPNN^h^, RBFNN^i^, FNN^j^, PNN^k^, and KNN	10-fold cross-validation (final evaluation)	—
Ai et al [93]	Questionnaire (standardized) and single items (frequency)	Questionnaire (nonstandardized)	—	Questionnaire (standardized)	Alzheimer disease	Transformation, missing imputation, and dimensionality reduction	RF and SVM^l^	Nested cross-validation	—
Allen [87]	Single items (environment)	Single items (environment)	Single items (sleep hours)	—	Obesity	Missing imputation and dimensionality reduction	RF and DT	2-fold cross-validation (final evaluation)	LIME^m^
Alshuraf et al [59]	Sensor (wearable)	Single items (habits)	—	Questionnaire (standardized)	Cardiovascular disease	Transformation, missing imputation, and dimensionality reduction	RF, DT, KNN, and NB	Leave-one-out cross-validation (LOOCV)	—
Birk et al [95]	Questionnaire (standardized)	Questionnaire (standardized)	—	—	Diabetes	Resampling and dimensionality reduction	RF	Hold-out test set	—
Bôto et al [62]	Single items (engagement)	Single items (habits)	Single items (sleep hours)	—	Lifestyle (diet)	Transformation and dimensionality reduction	DT	Not reported	—
Butkevičiūtė et al [40]	Sensor (wearable)	—	—	—	Lifestyle (physical activity)	Transformation	RF	5-fold cross-validation (final evaluation)	—
Cai et al [41]	Questionnaire (standardized)	—	—	—	Successful aging	Transformation and dimensionality reduction	RF, GBM^n^, and ANN	10-fold cross-validation (final evaluation)	—
Cheung et al [90]	Sensor (wearable)	—	—	Single items (stress level)	Lifestyle (physical activity)	Dimensionality reduction	RF and DT	Not reported	—
Chiang and Dey [102]	Sensor (wearable)	—	Sensor (wearable)	—	Blood pressure	Transformation, missing imputation, and dimensionality reduction	RF, GBM, MLPNN, LSTM-RNN^o^, and SVM	5-fold cross-validation (final evaluation) and online weighted-resampling	—
Cortés-Ibañez et al [73]	Questionnaire (standardized) and single items (frequency)	Questionnaire (standardized)	—	—	Cancer	Transformation, missing imputation, resampling, and dimensionality reduction	RF and SVM	Hold-out test set	—
Cortés-Ibañez et al [74]	Questionnaire (standardized) and single items (frequency)	Questionnaire (standardized)	—	—	Cancer	Transformation, resampling, and dimensionality reduction	RF, GBM, and SVM	5-fold cross-validation (final evaluation)	—
Dianati-Nasab et al [47]	Single items (engagement)	—	—	—	Cancer	Missing imputation and dimensionality reduction	RF, DT, XGBoost^p^, and ANN	10-fold cross-validation (final evaluation)	—
Faruqui et al [75]	Single items (frequency)	Single items (type of products)	—	—	Diabetes	Transformation and missing imputation	LSTM-RNN, ANN, and KNN	Hold-out test set	—
Gu et al [60]	Single items (frequency)	Single items (frequency)	Single items (sleep hours)	—	Infertility risk in women	Dimensionality reduction	RF, DT, BoostDT^q^, LightGBM^r^, and AdaBoost^s^	Hold-out test set	SHAP^t^ values
Guthrie et al [76]	Single items (frequency)	Single items (type of products)	—	—	Cardiometabolic disease	—	RF	Leave-one-out cross-validation (LOOCV)	SHAP values
Hu et al [77]	Single items (frequency)	—	Single items (sleep hours)	—	Cardiovascular disease	Missing imputation and dimensionality reduction	RF and BART^u^	Not reported	—
Hu et al [78]	Single items (frequency)	—	Single items (sleep hours)	—	Cardiovascular disease	Missing imputation and dimensionality reduction	RF, XGBoost, and BART	5-fold cross-validation (final evaluation)	—
Huang et al [63]	Single items (engagement)	—	Single items (sleep quality)	—	Cognitive function	Missing imputation and resampling	RF, BoostDT, XGBoost, and LSTM-RNN	Hold-out test set	SHAP values
Jin and Halili [67]	Single items (intensity)	—	Single items (sleep hours)	—	Mental health	Transformation, missing imputation, resampling, and dimensionality reduction	RF, DT, XGBoost, LightGBM, CatBoost^v^, Bagging^w^, HistGBM^x^, SVM, and MLPNN	Hold-out test set	SHAP values
Kim et al [91]	Questionnaire (standardized)	Questionnaire (standardized)	Questionnaire (standardized) and Single items (Sleep hours)	Questionnaire (standardized)	Quality of life	Transformation, Resampling	RF, DT, XGBoost, SVM, NB, KNN	6-fold cross-validation (final evaluation)	SHAP values
Kimura et al [104]	Sensor (wearable)	—	Sensor (wearable)	—	Alzheimer disease	Dimensionality reduction	SVM	5-fold cross-validation (final evaluation)	—
Kiss et al [64]	Single items (engagement)	—	Questionnaire (standardized) and single items (sleep hours)	Questionnaire (standardized)	Mental health	Transformation, missing imputation, and dimensionality reduction	XGBoost	Nested cross-validation	SHAP values
Li and Song [50]	Single items (engagement)	—	—	—	Cognitive function	Transformation	CNN^y^, Transformer, LSTM-RNN, GRU-Attention^z^, WaveNet, and RNN^aa^	10-fold cross-validation (final evaluation)	SHAP values
Lim et al [42]	Single items (intensity)	—	—	—	Osteoarthritis	Transformation, missing imputation, resampling, and dimensionality reduction	FFNN^ab^	Hold-out test set	—
Lim et al [10]	Single items (intensity)	—	Questionnaire (standardized) and single items (sleep quality)	Single items (Stress level)	Lifestyle (sleep)	Transformation, missing imputation, and dimensionality reduction	RF and DT	Hold-out test set	—
Lin et al [51]	Single items (engagement)	—	—	—	Loneliness	Transformation, Missing imputation, Dimensionality reduction	RF, DT, SVM, MLP, and KNN	10-fold cross-validation (final evaluation)	SHAP values
Liu et al [53]	Questionnaire (standardized)	—	—	—	Cardiovascular disease	Transformation	RSF^ac^	Hold-out test set	—
Luo et al [79]	Single items (frequency)	—	Single items (sleep hours)	Single items (stress level)	Social network addiction risk	Transformation and dimensionality reduction	RF	Hold-out test set	—
Luo et al [57]	Single items (intensity)	Questionnaire (standardized)	Single items (sleep hours)	Single items (stress level)	Chronic kidney disease	Missing imputation and dimensionality reduction	GBM	Hold-out test set	—
Luo et al [68]	Single items (intensity)	Single items (habits)	Single items (sleep hours)	—	Frailty	Missing imputation	XGBoost	10-fold cross-validation (final evaluation)	SHAP values
Majcherek et al [80]	Single items (frequency)	Single items (type of products)	Single items (sleep problems)	—	Mental health	Missing imputation	XGBoost	Not reported	SHAP values
Majcherek et al [65]	Single items (engagement)	Single items (habits)	—	—	Diabetes	Resampling	RF, DT, AdaBoost, CatBoost, HistGBM, LightGBM, XGBoost, KNN, NB, and Nearest Centroid	Hold-out test set	SHAP values
Matta et al [48]	Sensor (wearable)	—	—	—	Lifestyle (sleep)	Transformation	MLP	Hold-out test set	—
Moon and Woo [89]	—	Single items (habits)	Single items (sleep quality)	Single items (stress level)	Mental health	Transformation, missing imputation, resampling, and dimensionality reduction	RF and ANN	Not reported	—
Morris et al [88]	Questionnaire (standardized) and single items (environment)	Questionnaire (standardized) and single items (environment)	—	Questionnaire (standardized)	Cardiovascular disease	Missing imputation	RF and ANN	10-fold cross-validation (final evaluation)	SHAP values
Mousavi et al [66]	Single items (engagement)	Single items (frequency)	Single items (sleep hours)	—	Lifestyle (diet)	Dimensionality reduction	FFNN	Hold-out test set	—
Mun and Geng [81]	Single items (frequency)	—	Single items (sleep hours)	Questionnaire (standardized) and sensor (wearable)	Fatigue	Transformation, missing imputation, and dimensionality reduction	RF	10-fold cross-validation (final evaluation)	—
Nichols et al [61]	Questionnaire (standardized)	Questionnaire (standardized)	Questionnaire (standardized)	Questionnaire (standardized)	Lifestyle (stress)	Transformation, missing imputation, resampling, and dimensionality reduction	SVM	Hold-out test set	—
Oladeji et al [99]	Words (Google Trends)	Words (Google Trends)	—	—	Obesity	Dimensionality reduction	RF, GBM, and SVM	Out-of-sample	—
Park et al [49]	Single items (intensity)	—	—	—	Adverse health event	Dimensionality reduction	XGBoost	Hold-out test set	—
Park and Edington [82]	Single items (frequency)	Single items (type of products)	Single items (sleep hours)	Single items (stress level)	Diabetes	Missing imputation and resampling	MLPNN	Hold-out test set	—
Park [83]	Single items (frequency)	Questionnaire (standardized)	—	—	Visceral fat	Transformation, missing imputation, and dimensionality reduction	RF, XGBoost, and ANN	Hold-out test set	SHAP values
Pereira et al [92]	Questionnaire (standardized)	Questionnaire (standardized)	Questionnaire (standardized)	Questionnaire (standardized)	Mental health	Transformation and missing imputation	RF, XGBoost, and SVM	10-fold cross-validation (final evaluation)	—
Puterman et al [69]	Single items (intensity)	—	Single items (sleep problems)	—	Mortality	Transformation, missing imputation, and dimensionality reduction	RSF	Hold-out test set	—
Qasrawi et al [70]	Single items (intensity)	Single items (habits)	Single items (sleep hours)	—	Mental health	Missing imputation and dimensionality reduction	RF, DT, XGBoost, SVM, ANN, KNN	10-fold cross-validation (final evaluation)	—
Recenti et al [43]	Single items (frequency)	—	—	—	Lifestyle (physical activity)	Missing imputation and resampling	RF, GBM, and AdaBoost	10-fold cross-validation (final evaluation)	—
Recenti et al [44]	Single items (frequency)	—	—	—	Diabetes	Missing imputation and resampling	RF, GBM, and AdaBoost	10-fold cross-validation (final evaluation)	—
Ren et al [52]	Single items (engagement)	—	—	—	Cognitive function	Missing imputation, Resampling	RF, XGBoost, SVM	Hold-out test set	SHAP values
Ruiz et al [54]	Single items (engagement)	—	—	—	Depression	Not reported	DT	Not reported	—
Sandri et al [94]	Questionnaire (standardized)	Questionnaire (standardized)	Questionnaire (standardized)	—	Lifestyle (diet)	Transformation and resampling	RF, DT, XGBoost, CatBoost, HistGBM, and FFNN	Hold-out test set	SHAP values
Sathyanarayana et al [101]	Sensor (wearable)	—	Sensor (wearable)	—	Lifestyle (sleep)	Missing imputation	MLPNN, CNN, SETRNN^ad^, and LSTM-RNN	Hold-out test set	—
Shi et al [84]	Single items (frequency)	Single items (frequency)	Single items (sleep hours)	—	Osteoporosis	Transformation, missing imputation, resampling, and dimensionality reduction	RF, DT, SVM, and KNN	Hold-out test set	SHAP values
Staudenmayer et al [45]	Sensor (wearable)	—	—	—	Lifestyle (physical activity)	Transformation	RF, DT, ANN, and SVM	Leave-one-out cross-validation (LOOCV)	—
Stemmer et al [100]	Words (Twitter)	Words (Twitter)	—	—	Inflammatory bowel disease	—	RF, GBM, AdaBoost, and SVM	Hold-out test set	—
Su et al [56]	—	—	Questionnaire (standardized)	—	Resilience	Dimensionality reduction	RF, DT, and XGBoost	Not reported	SHAP values
Wallace et al [96]	Questionnaire (standardized)	—	Questionnaire (standardized) and single items (sleep hours)	—	Mortality	—	RSF	Not reported	—
Wallace et al [55]	—	—	Single items (sleep hours) and sensor (polysomnography)	—	Mortality	Missing imputation and dimensionality reduction	RF	External dataset	—
Wang et al [97]	—	Questionnaire (standardized)	Questionnaire (standardized)	—	Lifestyle (sleep)	Missing imputation, resampling, and dimensionality reduction	GBM, LightGBM, SVM, MLPNN, and KNN	10-fold cross-validation (final evaluation)	SHAP values
Xin and Ren [85]	Single items (frequency)	—	Single items (Sleep hours)	—	Mental health	Dimensionality reduction	RF	Hold-out test set	SHAP values
Zhang et al [86]	Single items (frequency)	Single items (habits)	Questionnaire (standardized)	Single items (stress level)	Lifestyle (sleep)	Resampling	RF, DT, XGBoost, SVM, ANN, and KNN	External dataset	SHAP values
Zhou et al [46]	Sensor (wearable)	—	—	—	Lifestyle (physical activity)	Transformation	SVM	Out-of-sample	—
Zhou et al [98]	Sensor (phone)	Sensor (phone)	—	—	Obesity	Missing imputation and dimensionality reduction	RF, GRF^ae^, and ANN	10-fold cross-validation (final evaluation)	—
Zhou et al [71]	Single items (intensity)	Single items (habits)	—	—	Psoriasis	Resampling	XGBoost	Hold-out test set	SHAP values

a ML: machine learning.

b XAI: explainable artificial intelligence.

c RF: random forest.

d ANN: artificial neural network.

e NB: naive Bayes.

f KNN: k-nearest neighbor.

g DT: decision tree.

h MLPNN: multilayer perceptron neural network.

i RBFNN: radial basis function neural network.

j FNN: fuzzy neural network.

k PNN: probabilistic neural network.

l SVM: support vector machine.

m LIME: local interpretable model-agnostic explanations.

n GBM: gradient boosting.

o LST-RNN: long short-term memory recurrent neural network.

p XGBoost: extreme gradient boosting.

q BoostDT: boost decision tree.

r LightGBM: light gradient boosting machine.

s AdaBoost: adaptive boosting.

t SHAP: Shapley Additive Explanations.

u BART: Bayesian additive regression trees.

v CatBoost: categorical boosting.

w Bagging: bootstrap aggregating.

x HistGBM: histogram-based gradient boosting machine.

y CNN: convolutional neural network.

z GRU-Attention: gated recurrent unit with attention.

aa RNN: recurrent neural network.

ab FFNN: feed-forward neural network.

ac RSF: random survival forest.

ad SETRNN: simple Elman-type recurrent neural network.

ae GRF: generalized random forest.

### Preprocessing

The preprocessing phase was divided into variable transformation, missing imputation, resampling, and dimensionality reduction. At least one of these preprocessing phases was reported by 59 (90.76%) studies (refer to Table 1 and Table S4 in Multimedia Appendix 1 for more details).

There were 26 (40%) studies that reported normalization or other arithmetic or statistical transformations of variables before the modeling phase [40,41,45,48,50,51,53,58,59,62,64,67,69,73-75,79,81,83,84,89,91-94,102]. Six (9.23%) studies recoded categorical variables into quantitative variables, 5 used one-hot encoding [10,53,64,67,102], and 1 used principal component analysis with quantile transformer scaler [42].

Missing data imputation was reported in 37 (56.92%) studies. Twelve papers simply removed cases with missing data [10,42-44,61,64,77,78,80,93,98,101], while others applied cutoff percentages for missing values (eg, 10% [87], 30 % [73], or >50% [58,72]), and 1 study removed observations with missing values in the output [102]. Techniques included single imputation (mean, median, or mode) [51,52,58,59,70,83,88,92], multiple imputation by chained equations [57,63,72,73], k-nearest neighbor [89,102], regression-based algorithm [57,81], random forest (RF)–based multiple imputation [55,69,84], the MissForest algorithm [67,68], imputation based on peers with similar health profile group [82], imputation using training data [104], and replacement of missing values with the last available data [75].

Resampling techniques were reported in 21 (32.30%) papers. Eighteen studies balanced the datasets using methods such as using the minority class as a reference, undersampling the majority class [42,61], or the synthetic minority oversampling technique (SMOTE) [43,44,52,60,63,67,71,72,84,86,89,91,94,95,97]. One paper compared the results of SMOTE against the adaptive synthetic algorithm [65]. Finally, 1 study stabilized variations in underrepresented outcome classes using bootstrap resampling [82]. Regarding cancer studies where cases were fewer than controls, 2 different strategies were applied to the same dataset: sample-size equalization by randomly grouping cancer-free participants based on the number of cancer survivors [74], while another study matched cases and controls by sex, age, and education level, then selected a random sample resulting in 50% cases and 50% controls [73].

Dimensionality reduction was used in 38 (58.46%) studies using 3 approaches. The first approach involved assessing the relationship between features and outcomes by removing redundant information [55,61,64,70,83,85,87,93,98,99]. Other methods included factor analysis [10] and principal component analysis [42,59,69]. The second approach optimized models to achieve lower prediction error [58,66,104]. The third approach involved automatic selection of predictors during model training [41,49,57,62,72-74,77,78,81,90,95,102]. For more information, refer to Table S4 in Multimedia Appendix 1.

### SL Models

#### Models Overview

In this section, the core components of ML models are described, beginning with problem formulation and algorithm families, followed by evaluation components. Depending on the purpose of the ML analysis, papers were grouped as classification or regression when the objective was prediction, and as feature selection when the goal was explanation [19]. Most studies (46/65, 70.77%) focused on classification, 9 (13.83%) on regression, 2 (3.1%) on both classification and regression, and 8 (12.30%) on feature selection.

**Figure 5.**
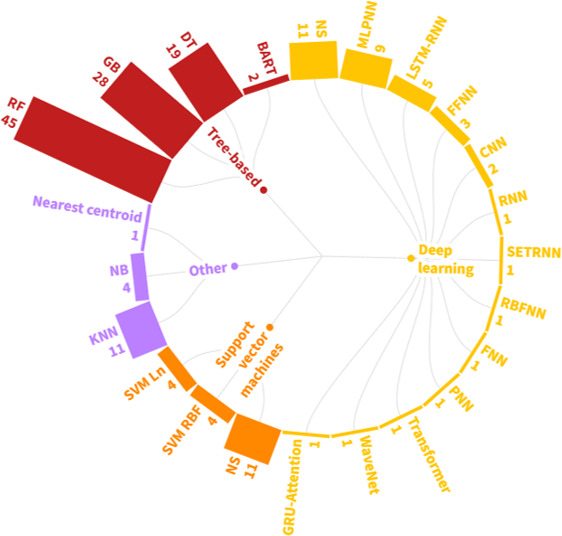
Machine learning families and algorithms taxonomy. BART: Bayesian additive regression trees; CNN: convolutional neural network; DT: decision tree; FFNN: feed-forward neural network; FNN: fuzzy neural network; GB: gradient boosting; GRU-attention: gated recurrent unit with attention; KNN: k-nearest neighbor; LSTM-RNN: long short-term memory recurrent neural network; MLPNN: multilayer perceptron neural network; NB: naive Bayes; NS: not specified; PNN: probabilistic neural network; RBFNN: radial basis function neural network; RF: random forest; SETRNN: simple Elman-type recurrent neural network; SVM Ln: support vector machine with linear kernel; SVM RBF: support vector machine with a radial basis function.

Six families of algorithms emerged from the studies: tree-based, deep learning, support vector machines, k-nearest neighbors, naïve Bayes, and nearest centroid. The approach adopted across 40 studies was a multimodel approach, with 29 of them incorporating algorithms from diverse algorithmic families. Figure 5 provides a comprehensive taxonomy of the specific algorithms implemented. The following subsections describe the application of specific algorithms within the 3 most used families (tree-based, deep learning, and support vector machines) in relation to the type of data used.

#### Tree-Based Algorithms

Tree-based algorithms were applied in 55 (84.61%) studies, covering all data types. RF was used in 45 (69.23%) out of 55 studies. Specifically, RF was implemented in 27 cross-sectional studies [40,43,44,47,51-53,56,60,65,70,72-74,77-79,84-87,89,91-95], 11 longitudinal studies [10,41,55,59,63,67,69,81,83,88,96], 3 time-series studies [45,76,102], 2 textual studies [99,100], 1 study with both cross-sectional and geographical data [98], and 1 study with both longitudinal and time-series data [90].

Different versions of the gradient boosting algorithm were performed in 28 studies, including gradient boosting machines, extreme gradient boosting, adaptive boosting, and light gradient boosting machine [41,43,44,47,49,52,56,57,60,63-65,67,68,70,71,74,78,80,83,86,91,92,94,97,99,100,102]. Finally, decision tree algorithms were implemented in 19 studies [10,45,47,51,54,56,58-60,62,65,67,70,84,86,87,90,91,94], and Bayesian additive regression trees in 2 studies [77,78] (Table 1; Figure 5).

#### Deep Learning Algorithms

Neural networks (NNs) are considered the cornerstone of deep learning algorithms. Various NN architectures were applied in 24 (36.92%) of the reviewed studies. A multilayer perceptron neural network was used in cross-sectional [51,58,97], longitudinal [67,82], and time-series data [48,101,102]. Long short-term memory recurrent neural network was applied to longitudinal [50,63] and time-series data [75,101,102]. Feed-forward neural networks were used for cross-sectional data [42,66,94]. Convolutional neural networks were used to analyze time-series [101] and cross-sectional data [50]. Simple Elman-type recurrent neural networks were applied in a time-series study [101]. Radial basis function neural networks, fuzzy neural networks (FNNs), and probabilistic neural networks were used in a cross-sectional study [58]. In addition, Transformer, gated recurrent unit with attention, WaveNet, and RNNs were used to analyze longitudinal data [50]. In contrast, 11 studies did not specify the artificial neural network architecture used [41,45,47,70,72,75,83,86,88,89,98] (Table 1; Figure 5).

#### Support Vector Machine Algorithms

Support vector machine (SVM) algorithms were used in 19 (27.08%) studies, applied across various data types. SVM was implemented in 11 cross-sectional studies, 2 longitudinal studies, 3 time-series studies, and 2 studies that analyzed textual data. Configurations included support vector machine with a radial basis function [61,70,102,104] or a support vector machine with linear kernel [51,73,93,100]. Eleven studies did not report the type of kernel used [45,46,52,67,74,84,86,91,92,97,99] (Table 1; Figure 5).

### Evaluation

Final model evaluation procedures were explicitly reported in 57 (87.69%) reviewed studies, with the hold-out test set being the most applied strategy. These included a hold-out test set [10,42,48,49,52,53,57,60,61,63,65-67,69,71-73,75,79,82-85,94,95,100,101], k-fold cross-validation (final evaluation) [40,41,43,44,47,50,51,58,68,70,74,78,81,87,88,91,92,97,98,104], nested cross-validation [64,93], leave-one-out cross-validation (for small datasets with n<150) [45,59,76]. Two studies used external datasets for model performance assessment [55,86]. For time-series data, 2 studies divided the dataset based on the time of acquisition, keeping an out-of-sample dataset for model evaluation [46,99], while 1 study [102] used 5-fold cross-validation in an offline setting followed by an online weighted resampling methodology to address drift.

Among the 11 studies that addressed regression problems, the reported evaluation metrics included mean absolute error [44,75,87,102], mean squared error [44,68,75], root-mean-square error [44,45,68,78,98,99,102], mean absolute percentage error [102], and coefficient of determination (R^2^) [44,68,79,81,98,99].

For classification problems, reported evaluation metrics included specificity and sensitivity (recall) [41-44,47,48,58,63,66,72,76,82,85,86,91,93,95,104], precision and recall [40-43,48,61,70,80,86,100,101], the confusion matrix [42,48,80], error rate as the proportion of misclassified observations (1 – accuracy) [48,58,72,90], Cohen κ [58,72], *F*_1_-score [41,43,49,59,70,80,86,91,100,101,104], and model training time [58]. The most frequently used metrics were accuracy [10,41-45,47-49,58,59,61,63,66,70,72,80,82,83,85,86,88,91,93,101] and area under the receiver operating characteristic curve [41-43,46-49,58,59,63,70,72-74,76,83,85,86,91,93,95,100,101,104] (Table S5 in Multimedia Appendix 1). Additionally, 1 epidemiologic study used the Brier score to assess cardiovascular mortality [53].

### Explainability

**Figure 6.**
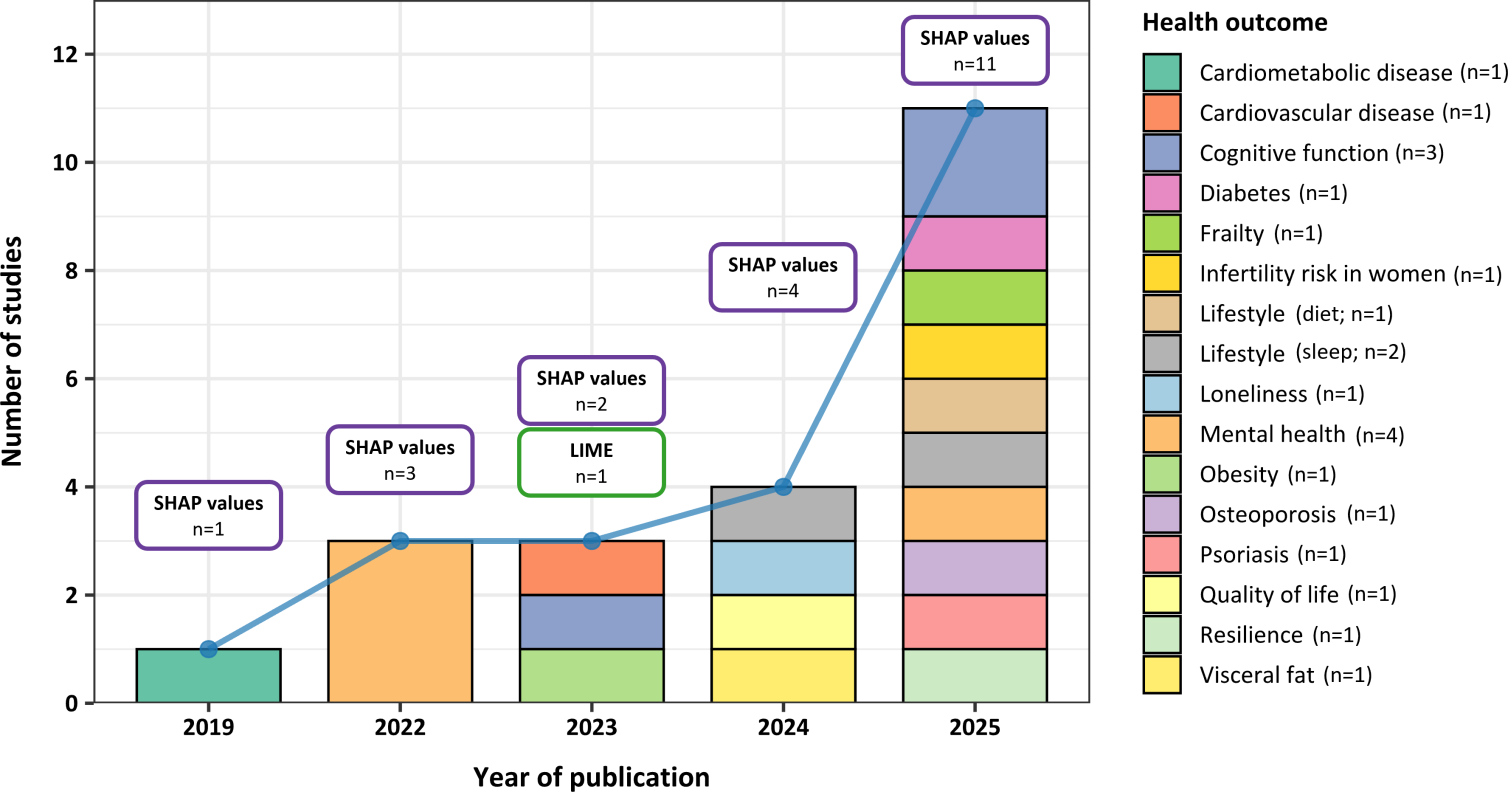
Bar plot depicting the application of explainable artificial intelligence (XAI) algorithms in different years per health outcome (N=22). LIME: local interpretable model-agnostic explanations; SHAP: Shapley Additive Explanations.

To enhance explainability, 22 (33.84%) studies implemented specific XAI methods to clarify the contribution of each predictor and the direction of the relationship with the outcome. Of these, 21 studies used Shapley Additive Explanations (SHAP) values, while 1 used local interpretable model-agnostic explanations (LIME). As shown in Figure 6, XAI methods, particularly SHAP values, have been applied across different health domains, showing a remarkable increase in use in 2025. The most common visualization methods were beeswarm and bar plots, with the contribution of each feature ranked along the y-axis, placing the most important feature at the top of the plot. The number of features plotted ranges from 6 to 50, with 20 being the most common (refer to Table 2).

**Table 2.** Summary of explainable artificial intelligence use by health outcome and healthy lifestyle components.

Study	Health outcome	Total number of features (features ranked)	Healthy lifestyle components (category, position, features ranked)	XAI^a^ (visualization)
Allen [4]	Obesity	64 (10)	Physical activity (physical inactivity, 1, 10); diet (food insecurity, unranked, n/a); sleep (sleep hours, unranked, n/a); stress (n/ac^b^, n/a^c^, n/a).	LIME^d^ (waterfall plot)
Gu et al [16]	Infertility risk in women	39 (10)	Physical activity (physical activity health score, unranked, n/a); diet (diet health score, 10, 10); sleep (sleep health score, unranked, n/a); stress (n/ac, n/a, n/a).	SHAP^e^ values (beeswarm plot, bar plot, and dependency plot)
Guthrie et al [17]	Cardiometabolic disease	13 (13)	Physical activity (minutes of physical activity, 8, 13); diet (plant-based meal, 6, 13); sleep (n/ac, n/a, n/a); stress (n/ac, n/a, n/a).	SHAP values (beeswarm plot)
Huang et al [20]	Cognitive function	20 (20)	Physical activity (exercise, 10, 20); diet (n/ac, n/a, n/a); sleep (sleep quality, 8, 20); stress (n/ac, n/a, n/a).	SHAP values (beeswarm plot and bar plot)
Jin and Halili [21]	Mental health	21 (21)	Physical activity (intensity, unranked, n/a); diet (n/ac, n/a, n/a); sleep (sleep hours, 1, 21); stress (n/ac, n/a, n/a).	SHAP values (bar plot)
Kim et al [22]	Quality of life	21 (20)	Physical activity (transport-related, 4, 20; physical activity score, 7, 20); diet (eating index, 15, 20); sleep (sleep quality, 2, 20; sleep hours, 6, 20); stress (stress level, 1, 20).	SHAP values (beeswarm plot)
Kiss et al [24]	Mental health			SHAP values (bar plot)
		Positive affect: 231 (20)	Physical activity (doing outdoor activities, 3, 20; duration of sitting, 6, 20; frequency of walking, 7, 20); diet (n/ac, n/a, n/a); sleep (sleep disorder, 8, 20); stress (coping strategies, 9, 20).	
		Perceived stress: 228 (20)	Physical activity (engagement, 12, 20); diet (n/ac, n/a, n/a); sleep (sleep hours, 7, 20); stress (coping strategies, 5, 20).	
		Anxiety: 228 (20)	Physical activity (engagement, unranked, n/a); diet (n/ac, n/a, n/a); sleep (sleep hours, 15, 20); stress (coping strategies, 10, 20).	
		Depressive symptoms: 240 (20)	Physical activity (engagement, 14, 20); diet (n/ac, n/a, n/a); sleep (sleep hours, 16, 20); stress (coping strategies, 9, 20).	
Li and Song [25]	Cognitive function	20 (20)	Physical activity (sport social capital index, 3, 20); diet (n/ac, n/a, n/a); sleep (n/ac, n/a, n/a); stress (n/ac, n/a, n/a).	SHAP values (beeswarm plot, heat map, temporal analysis, and dependency plot)
Lin et al [28]	Loneliness	15 (15)	Physical activity (exercise, 7, 15); diet (n/ac, n/a, n/a); sleep (n/ac, n/a, n/a); stress (n/ac, n/a, n/a).	SHAP values (beeswarm plot)
Luo et al [33]	Frailty			SHAP values (beeswarm plot and dependency plot)
		US cohort: 121 (20)	Physical activity (play sports and exercise, 8, 20; moderate physical activity, 16, 20); diet (n/ac, n/a, n/a); sleep (sleep problems, 4, 20); stress (n/ac, n/a, n/a).	
		UK cohort: 125 (20)	Physical activity (vigorous physical activity, 4, 20; moderate physical activity, 5, 20); diet (fruit consumption, 19, 20); sleep (sleep problems, 2, 20; sleep duration, 6, 20); stress (n/ac, n/a, n/a).	
		China cohort: 94 (20)	Physical activity (n/ac, n/a, n/a); diet (n/ac, n/a, n/a); sleep (sleep problems, 5, 20; sleep duration, 8, 20); stress (n/ac, n/a, n/a).	
Majcherek et al [34]	Mental health	26 (24)	Physical activity (sport, 6, 24; walking time, 11, 24); diet (vegetable portion, 13, 24; fruit portion, 14, 24); sleep (n/ac, n/a, n/a); stress (n/ac, n/a, n/a).	SHAP values (dependency plot)
Majcherek et al [35]	Diabetes	22 (6)	Physical activity (regular physical activity, unranked, n/a); diet (habits, unranked, n/a); sleep (n/ac, n/a, n/a); stress (n/ac, n/a, n/a).	SHAP values (dependency plot)
Morris et al [38]	Cardiovascular disease	50 (50)	Physical activity (availability of outdoor activities, 5, 50); diet (available favorable food stores, 14, 50); sleep (n/ac, n/a, n/a); stress (global stress, 18, 50).	SHAP values (bar plot)
Park et al [43]	Visceral fat	32 (20)	Physical activity (frequency, unranked, n/a); diet (high rice consumption, 2, 20; Asian-style balanced diet, 6, 20); sleep (n/ac, n/a, n/a); stress (n/ac, n/a, n/a).	SHAP values (beeswarm plot and bar plot)
Ren et al [51]	Cognitive function	39 (20)	Physical activity (exercise, 16, 20); diet (n/ac, n/a, n/a); sleep (n/ac, n/a, n/a); stress (n/ac, n/a, n/a).	SHAP values (beeswarm plot, bar plot, and force plot)
Sandri et al [53]	Lifestyle (diet)			SHAP values (beeswarm plot)
		Mediterranean diet: 41 (20)	Physical activity (sport, 8, 20); diet (fish consumption, 1, 20); sleep (sleep quality, unranked, n/a); stress (n/ac, n/a, n/a).	
		Intermittent fasting: 41 (20)	Physical activity (sport, 3, 20); diet (fish consumption, 1, 20); sleep (sleep quality, 14, 20); stress (n/ac, n/a, n/a).	
		Vegan diet: 41 (20)	Physical activity (sport, 5, 20); diet (fish consumption, 1, 20); sleep (sleep quality, 9, 20); stress (n/ac, n/a, n/a).	
		Vegetarian diet: 41 (20)	Physical activity (sport, 5, 20); diet (fish consumption, 1, 20); sleep (sleep quality, unranked, n/a); stress (n/ac, n/a, n/a).	
Shi et al [55]	Osteoporosis	45 (20)	Physical activity (physical activity health score, 16, 20); diet (diet health score, unranked, n/a); sleep (sleep health score, 5, 20); stress (n/ac, n/a, n/a).	SHAP values (beeswarm plot, waterfall plot, and force plot)
Su et al [58]	Resilience	12 (4)	Physical activity (n/ac, n/a, n/a); diet (n/ac, n/a, n/a); sleep (sleep disturbance, 3, 4); stress (n/ac, n/a, n/a).	SHAP values (beeswarm plot, bar plot, and dependency plot)
Wang et al [60]	Lifestyle (sleep)	7 (7)	Physical activity (n/ac, n/a, n/a); diet (nutritional status, 3, 7); sleep (outcome, n/a, n/a); stress (n/ac, n/a, n/a).	SHAP values (beeswarm plot, bar plot, and waterfall plot)
Xin and Ren [62]	Mental health			SHAP values (beeswarm plot and bar plot)
		Rural older adults: 55 (20)	Physical activity (exercise, 19, 20); diet (n/ac, n/a, n/a); sleep (sleep hours, 13, 20); stress (n/ac, n/a, n/a).	
		Urban older adults: 55 (16)	Physical activity (exercise, unranked, n/a); diet (n/ac, n/a, n/a); sleep (sleep hours, 6, 16); stress (n/ac, n/a, n/a).	
Zhang et al [63]	Lifestyle (sleep)	20 (10)	Physical activity (sedentary time, 5, 10); diet (vegetable consumption, 3, 10); sleep (outcome, n/a, n/a); stress (stress score, 1, 10).	SHAP values (beeswarm plot and dependency plot)
Zhou et al [63]	Psoriasis	150 (20)	Physical activity (intensity, 17, 20); diet (habits, unranked, n/a); sleep (n/ac, n/a, n/a); stress (n/ac, n/a, n/a).	SHAP values (beeswarm plot)

a XAI: explainable artificial intelligence.

b n/ac: not applicable.

c n/a: not available.

d LIME: local interpretable model-agnostic explanations.

e SHAP: Shapley Additive Explanations.

Because the nonlifestyle features included in each model vary substantially across studies, the ranking reported in Table 2 should be interpreted in relative terms. When a lifestyle behavior appears among the top-ranked features, this indicates that it contributed more strongly to the model than nonlifestyle variables included in the analysis. Conversely, lifestyle components appearing in lower positions acted as secondary predictors.

In a study on cardiometabolic disease [76], the ML solution was first explained at the individual participant level to provide specific behavioral feedback, and then at the group level to reveal the ranking of features for succeeding in behavioral changes. In both models, physical activity and diet variables were among the top contributors. However, in another study [65], neither regular physical activity nor diet habits were among the top 6 variables for predicting diabetes in adults.

In 4 mental health studies [64,67,80,85], SHAP values were used to rank the contribution of each feature. In 3 of the 4 studies, lifestyle variables were among the top contributors. In [80], physical activity and fruit and vegetable consumption; in [64], sleep variables were the top predictors of stress in young adolescents during the COVID-19 pandemic; and in [85], sleep duration was identified as important for predicting depression. However, in [67], the intensity of physical activity was unranked because it was excluded from the XAI analysis during a feature selection step prior to modeling. In the same study, sleep hours were the most important variable for predicting depression among adults. Regarding mental health studies, psychological resilience was assessed among medical students [56], with sleep disturbance being a key factor affecting their resilience.

Three studies focusing on older adults in China had cognitive function as their outcome [50,52,63]. In these studies, physical activity variables were ranked as top predictors. Additionally, in 1 of these studies [63], sleep quality was ranked as the eighth top feature out of 20. Furthermore, when loneliness was assessed among older adults from China [51], exercise was ranked as the seventh variable out of 15.

In a study predicting quality of life [91], stress, sleep quality, and physical activity emerged as the strongest predictors, with the eating index appearing in the top 15 variables.

Two studies focused on sleep as the specific outcome. In [86], the SHAP values ranked stress score, vegetable consumption, and sedentary time among the top 5 variables for predicting sleep disturbance. In [97], nutritional status was the third most important variable for predicting the risk of sleep disorders in older adults.

In [88], “favorable” food stores and global stress were identified as the top variables for predicting incidence of cardiovascular disease, with the availability of outdoor activities ranking in the top 5. In a longitudinal study investigating the association of diet with long-term reduction in waist circumference, SHAP values highlighted the importance of high-quality components in reducing visceral fat [83]. This study also measured exercise with a single item of frequency, which was not included in the top ranking. Regarding a diet study [94], the adoption of different diets in the Spanish population was assessed, with fish consumption positioned as the most important variable for all diets studied (Mediterranean, intermittent fasting, vegan, and vegetarian). In the same study, practicing sport was ranked among the top variables across the different diets. However, sleep quality was only included in the ranking for intermittent fasting and the vegan diet.

Physical inactivity emerged as the most important feature in explaining county-level obesity using LIME [87]. In this study, the food environment and insufficient sleep, both measured as single items, were not included as top predictors of obesity prevalence.

In a cross-national study assessing frailty [68], sleep variables were among the 20 most important variables across all the cohorts studied. Physical activity was included in the US and UK cohorts and was also ranked in the top 20 variables. In the UK cohort, fruit consumption was the 19th out of 20 key contributors to frailty.

One study on osteoporosis [84] used the Life’s Essential 8 scores for physical activity, diet, and sleep. Sleep and physical activity scores were among the 20 most important variables, but the diet health score was unranked. Similarly, the Life’s Essential 8 scores were used to determine infertility risk in women [60], with the diet score being the only one ranked among the top 10 variables.

Finally, 1 study combined lifestyle factors with metabolites associated with psoriasis [71], with physical activity intensity ranked among the 20 key factors out of 150 variables, whereas dietary habits were not ranked.

### Software to Implement ML Models

Of the included papers in the review, 23 (35.38%) used R software (R Core Team) [105] for data analysis (Table 3). The R packages used were data.table for data manipulation, tidyverse [106] as a general package for data science, Multivariate Imputation by Chained Equations [107] for missing data imputation, missMDA for performing multiple imputation with principal component analysis, FactoMineR for exploratory data analysis and principal component analysis, Boruta [108] for feature selection through a wrapper algorithm, caret (Classification and Regression Training) [109] for creating models, randomForest for RF analysis, randomForestSCR for RF for survival, regression, and classification analysis, rpart for recursive partitioning and regression trees, xgboost for extreme gradient boosting, bartMachine for Bayesian additive regression trees, kernlab and e1071 for support vector machines, survival for survival analysis, lime for local interpretable model-agnostic explanations, and finally, SuperLearner [110] to choose the optimal learner for a given prediction problem with a k-fold cross-validation algorithm.

**Table 3.** Softwares used in the reviewed studies to perform machine learning (ML) algorithms.

Software used	Number of studies	Study references
R (R Core Team)	n=23	[41,45-47,49,54,55,69,72-74,77,78,80,87,90,93,95-97,99,101,111]
Python (Python Software Foundation)	n=24	[42,50,52,53,56,58,61,64,65,67,70,76,79,81,83,84,86,88,89,91,98,100,102,104]
R and Python	n=4	[57,63,68,71]
SPSS (IBM Corp)	n=2	[62,92]
MATLAB (The MathWorks Inc)	n=2	[48,66]
KNIME [112]	n=2	[43,44]

In contrast, 24 (36.92%) studies developed the models in Python (Python Software Foundation), using the following libraries: Scikit-learn, used in all studies for predictive data analysis; pandas, for manipulating tabular data; NumPy, for mathematical functions; Keras and TensorFlow, for implementing deep learning; lightGBM, for performing light gradient boosting machine; SHAP, to explain ML solutions; creme, for online ML; Bayesian optimization, as a global optimization package to find the maximum value of an unknown function in as few iterations as possible; imbalanced-learn, to combine either undersample or oversample methods; and TextBlob, emoji, nltk, and profanity, for processing and analyzing textual data*.*

Finally, 4 studies used both Python and R, and 6 studies used other software programs such as SPSS, MATLAB, and KNIME. Eight papers did not report the software used [10,40,59,60,75,82,85,94].

## Discussion

### Overview

This scoping review of 65 studies provides the current state of the application of supervised ML algorithms for the analysis of lifestyle data. The increase in studies in this field since 2019 indicates that it is a noteworthy area of study. The diversity in the sample origin, alongside the accessibility to new AI tools and novel methods for monitoring health outcomes (eg, wearables), denotes global attention to lifestyle. This section addresses the methodological shortcomings found in the reviewed studies.

### About Data Acquisition

In relation to lifestyle data, we found that most studies adopted a multidomain approach, integrating more than just a single component. This strategy enhances and facilitates a more comprehensive understanding of health problems related to the 4 lifestyle domains considered in this review: physical activity, diet, sleep, and stress. The distribution of lifestyle domains identified here was similar to that reported in a previous scoping meta-review [113], although we observe that sleep has gained prominence in recent years, now reaching a level comparable to diet. Although these results highlight the growing recognition of the interrelated nature of lifestyle behaviors, the imbalance in the distribution of these factors limits the capacity of current studies to fully model and understand the interaction among the 4 lifestyle components and their combined effects on health.

Concerning the data acquisition process, over half of the studies acquired their own data. This acquisition process implies control over variables and reduces the time required for cleaning [114]. Interestingly, both self-acquired datasets and those sourced from private or public health repositories demonstrated gender parity in the analyzed datasets. However, we detected a major limitation in this part of the process regarding the data acquisition methodology. In most studies, data were collected through single items, such as regular physical activity (response “yes” or “no”) [58] or usual time of waking up and going to bed [62], resulting in low representativeness of the construct being measured. The result of this acquisition method is high heterogeneity in measures, which hinders their generalizability. Therefore, the quality of data must be one of the challenges to be addressed, and specifically, the consistency in measures [115,116].

Nevertheless, the current accessibility and precision of health sensors such as wearables [117] and the Internet of Things [118] may contribute to transferability and actionability in the population [119]. The growth in technology allows the integration of different data forms as well as more objective measures of lifestyle, substantially reducing the impact of retrospective bias by tracking real-time data in an ecological situation [120]. Therefore, merging questionnaires and sensor data may be the key to identifying relationships between lifestyle measurements and personalizing interventions or changes in specific behaviors. This integration would include physiological, psychological, and behavioral factors, which are the most common analysis types in the ML community to extract clinical insights [121].

### About Characteristics of ML Models

Regarding the analysis of ML, 2 different approaches emerged in the reviewed studies: 1 focused on prediction through classification and regression problems, and the other focused on interpretability through feature selection. The first is already an acknowledged approach, while the second typically constitutes an important component of the ML process, specifically during the preprocessing stage. However, feature selection studies do not use model evaluation metrics, which can limit their statistical validity and the generalizability of results. Remarkably, the family of ML algorithms most closely related to feature selection is tree-based because it provides indices of the importance of each variable. Although most papers in this scoping review combined different families of algorithms and compared their results, the most common model family was tree-based, which was applied for each data typology identified. Specifically, RF is the most used algorithm, which may be due to its robustness in handling missing values, the consideration of complex interaction in the data [122], and its lower sensitivity to variable scales [123]. Despite the benefits of RF, the underusage of DL algorithms represents a critical missed opportunity for robustly analyzing complex and multimodal data. In this review, DL algorithms were underused for lifestyle data. This result may reflect a gap in expertise or access to computational resources among lifestyle researchers, potentially limiting the application of more complex models. With ongoing advances in computational power and algorithmic efficiency, it is expected that the use of DL algorithms will become more widespread in the near future [124].

Regarding the preprocessing stage, most studies detailed some phases of the process, but there is no consensus on the description of this stage of ML. Variable transformation is a crucial step for certain algorithms, particularly for SVM and specific architectures of DL that exhibit sensitivity to the raw form of the variables. In this review, 12 out of 19 studies that performed the SVM algorithm, and 13 out of 26 studies focused on DL, reported variable transformation. Additionally, it is worth noting that these algorithms cannot handle missing values, requiring imputation before the modeling phase. Among the SVM studies, 11 out of 19 reported techniques for missing imputation, and 17 out of 26 DL studies explicitly addressed this. In contrast, tree-based algorithms are less sensitive to variable scales and missing values, yet incorporating these feature engineering steps could enhance model performance [123]. It is noteworthy that the preprocessing steps, specifically how missing values are addressed, have been identified as a potential concern for transparency. This procedural aspect could introduce sampling biases, thereby influencing the generalizability and comprehension of the dataset context [125].

Resampling techniques, aiming to balance the dataset, are commonly implemented in classification problems. SMOTE has been the most widely used technique in this review, especially because it achieves better results than a simple undersampling of the majority class. In the health domain, imbalanced datasets are common, and SMOTE oversamples the minority class with synthetic examples and randomly undersamples the majority class to balance the dataset [126].

Finally, dimensionality reduction enables capturing the most relevant information for the outcome while eliminating noise and redundant information. In this review, dimensionality reduction was the most frequent preprocessing step, appearing in 38 studies. Notably, not only could SVM and DL models benefit from removing irrelevant predictors, but also in tree-based algorithms, dimensionality reduction minimizes model complexity, resource consumption, and data acquisition costs [123].

The division of the original dataset is an essential step for assessing the performance of the ML solution. In this review, 8 studies did not report how they split their data to assess the model, which denotes a lack of generalization of their results. This omission represents a common issue in ML research that should be carefully addressed to minimize bias [127]. Train and test division, also known as “hold out,” is a method with considerable variability due to the use of a unique random data distribution [127]. Therefore, other methods might be more suitable. For example, leave-one-out cross-validation, which trains the model on “n – 1” observations and makes predictions on the remaining one. Although effective for small datasets, it is computationally intensive with large datasets [128]. K-fold cross-validation involves randomly dividing the original dataset into k groups. K-fold cross-validation not only offers computational advantage over leave-one-out, but also gives more accurate estimations due to the bias-variance trade-off [128]. In time-series data, only 1 paper [102] introduced a different form of data split, considering the dependencies of the entire series. Ideally, this type of data should be treated with a method called rolling forecast origin resampling, which estimates the model with historical data and evaluates it with the most recent data [129]. In other words, the training set should ideally comprise observations that occurred before those in the test set; however, this method was not found in this review.

Regarding the evaluation of ML models, the choice of metrics depends on the nature of the problem, whether regression or classification. Specific evaluation metrics tailored to each problem are crucial for correct evaluation, aligning with the priorities and needs of each field. For instance, in medical studies where the cost of treatment in terms of health is high, it becomes crucial to identify true patients over false positives. In contrast, if the treatment has minimal side effects and has demonstrated benefits, sensitivity might not be as important as specificity. It is worth highlighting that in the field of data science, precision and recall are more commonly used, whereas in medical fields, specificity and sensitivity are more prevalent [18]. These differences may cause misunderstandings between the 2 domains.

### About Explainability Methods

Regarding model explainability, 22 studies incorporated a dedicated step in the ML process for explainability. SHAP values and LIME were the only XAI methods applied to lifestyle data, and these 2 methods were the most common in a recent systematic review on XAI methods [130]. XAI-related studies in this review were published since 2019, with a notable increase in the number of publications in 2025, comprising half of the papers. This exponential growth was also found in [27,28], where the trend in published papers occurred between 2016 and 2022. Thus, our review demonstrates this exponential distribution in the health and behavioral sciences, where XAI methods are gaining prominence.

Although tree-based algorithms, especially decision trees, are known for facilitating interpretation, SHAP values can be applied to any type of model [131]. The adoption of XAI in lifestyle studies remains low (33.84%). One possible explanation for this is that explainability algorithms are often not integrated into a standard ML pipeline, thereby increasing the technical complexity of the workflow. However, some efforts are being made by R and Python developers to incorporate XAI algorithms into pipelines using libraries such as H2O [132].

In this review, lifestyle components (physical activity, diet, sleep, and stress) consistently appeared among the top-ranked features in models using XAI techniques, highlighting their substantive contribution relative to nonlifestyle variables. These findings align with prior research emphasizing the integration of diverse lifestyle components [133,134]. However, the level of interpretability achieved also depends on the quality of data used in the models, which in some studies did not meet expected standards. For instance, in a study where the focus was on obesity, the diet component was not among the top-ranked features [87]. In this county-level study, a food environment index was measured as a single item, potentially inadequately representing the diet component of lifestyle.

Therefore, integrating XAI methods into the ML process could enable tailored interventions based on model results, provided that measures are collected accurately. Furthermore, the adoption of XAI algorithms contributes to increased trust and verification of the fairness of the models. This approach can also facilitate the translation of findings to stakeholders and health systems, thereby enhancing transparency, promoting the adoption of models in society, and supporting informed decision-making [27].

### About the Software for Implementing ML Models

Competition between Python and R for ML software dominance in data science is currently intense. Both Python and R are freely distributed, object-oriented software with large and active communities. Python, as a programming language, offers specific implementations through libraries tailored for statistical analysis, including ML and DL. In contrast, R is a statistical software that integrates fundamental statistics into its base functionalities. While Python requires libraries for each stage of analysis, its well-established libraries streamline the process. On the other hand, R faces challenges due to its heterogeneous libraries, which hinder replicability and require expertise in varying syntax across packages. To address this concern, the meta-package tidymodels (Max Kuhn and Hadley Wickham) resolves these issues by integrating all necessary packages for each ML step, using a unified syntax. Additionally, tidymodels integrates user-friendly interfaces and promotes good methodological practice, thereby preventing user errors [135]. Conversely, Python presents a preferable environment for DL with the TensorFlow and PyTorch frameworks. In this regard, the possibility of developing ML projects on powerful computational cloud-based platforms, such as Google Colaboratory (also referred to as Google Colab) [136], offers Python a remarkable advantage over R local environments.

### Methodological and Reporting Guidelines and Checklist

Based on the review’s results and to enhance transparency and replicability in multidisciplinary sciences [137], we provide comprehensive methodological and reporting guidelines and a checklist for ML projects. Although various studies have proposed guidelines and checklists [138,139], the rapid expansion of ML algorithms in health domains necessitates iterative evaluation to incorporate new steps into the ML research workflow. The guidelines and checklist (Checklist 2) are based on the 5 stages of the ML workflow, as depicted in Figure 2, with added software tools.

#### Data Acquisition

The integration of multidomain data enhances the comprehension of real-world problems. Using appropriate methods to collect data ensures representativeness. We recommend the use of standardized questionnaires and validated sensors. Regarding health repositories, we recommend providing information about data characteristics such as gender distribution, sample size, and variable descriptions [140].

#### Preprocessing

Reporting the preprocessing methods used in the data analysis is particularly crucial for ensuring replicability. While preprocessing contributes to improving data quality, different preprocessing methods can lead to different results. We propose the following recommendations for each preprocessing step, although not all steps need to be performed in every ML project.

##### Transformation

Categorical data should be encoded using methods such as one-hot encoding and dummy variables. Continuous data should be transformed using normalization or rescaling of features with different units to ensure algorithm performance, particularly for those sensitive to the raw form of variables [123].

##### Missing Imputation

Some algorithms cannot handle missing data and require imputation before modeling. Depending on the number of observations and the data distribution, imputation with the mean, median, or mode is typical. For time-series data, imputation with the last or next observation is preferred, though rolling statistics imputation or interpolation may offer better solutions [141].

##### Resampling

Imbalanced datasets can bias models, resulting in poor performance on underrepresented classes [142]. While SMOTE is an effective technique for handling imbalanced datasets, it is not without its limitations. However, when the class imbalance ratio is extremely high, SMOTE can potentially bias the model performance by overfitting the minority class. This issue is particularly pronounced in datasets containing noise, as synthetic observations may replicate these artifacts. To mitigate these challenges, recent studies have proposed tree algorithms, which have shown effectiveness in handling class imbalance [143]. Furthermore, due to their robustness, the use of tree-based algorithms is increasingly recommended when working with class imbalance [144].

##### Dimensionality Reduction

Removing noise from the dataset and retaining features directly related to the outcome can enhance both data acquisition and modeling efficiency. Removing correlated features is particularly beneficial [123].

### Modeling

Our guidelines focused on SL algorithms for classification and regression problems. The choice of algorithms depends on the measurement of the outcome. We recommend using multiple algorithms to compare results and select the best fit, given that there is significant variability across problems [145]. Additionally, comparing different families of algorithms is also advisable, as some improvements exist within the same family. When it comes to replicability and transparency, reporting algorithm hyperparameters is crucial in ML problems, as different configurations can yield varying results [146].

### Evaluation

Avoiding overfitting requires dividing the original dataset appropriately. This step is fundamental in ML implementation and should be considered in every study to ensure that the extracted insights are reliable and generalizable. This division depends on data typology; we identified 3 different typologies relevant at this stage, defined by whether time is an implicit factor in the data acquisition process.

#### Cross-Sectional

Data are collected at a single point in time, with no temporal dependencies. Leave-one-out cross-validation is effective for small datasets (n<150) typically seen in life sciences; the computational complexity increases [147]. K-fold cross-validation is recommended for large datasets to balance the bias-variance trade-off [128]. Cross-validation with bootstrap resampling can also be used to evaluate the performance of the models and estimate CIs of performance metrics [148].

#### Longitudinal

In longitudinal studies, researchers collect repeated measures, potentially with dependencies between observations and high correlation that can bias the model [135]. Data should be divided by grouping individual participants’ information. The methods used are similar to those for cross-sectional data, but with consideration of partitioning.

#### Time Series

Time series data are a sequence of data points in chronological order. Rolling forecast origin resampling is suitable for this data [129]. The training set should include observations occurring before those in the test set.

#### Evaluation Metrics

Appropriate metrics should be selected based on the type of ML problem (regression or classification) [149] and the characteristics of the study field [18]. It is also recommended to compute evaluation metrics repeatedly across cross-validation samples and to apply nonparametric tests, such as the Wilcoxon signed-rank test and the Friedman test, to assess model performance [149]. For imbalanced datasets, performance metrics such as balanced accuracy and *F*_1_-score are recommended. For example, in an imbalanced dataset, a model may achieve high accuracy by predominantly predicting the majority class. In contrast, balanced accuracy assigns equal weight to each class regardless of its frequency, providing a more informative evaluation of model performance [150].

### Explainability

#### Explainability Overview

Reporting the method of explainability used in the ML projects is essential. It is important to distinguish between interpretability and explainability. While explainability refers to understanding the effect of each feature on the original model, interpretability involves deriving actionable insights from the model’s prediction. Although tree-based methods include importance metrics, they do not indicate the direction of relationships with the outcome. Incorporating explainable resources such as SHAP values [151] or LIME [152] enhances the interpretability of the results, providing both actionability and transparency, and transforming black boxes into glass box models. The H2O package in R offers XAI algorithms that are compatible with the framework tidymodels, enabling a unified workflow for modeling and explainability. For a more detailed taxonomy of XAI packages in R, we refer the reader to [153].

Additionally, the choice of appropriate visualization methods and the number of features displayed are crucial for ensuring comprehensive results and supporting fine-grained decision-making. Regarding the number of features, we recommend visualizing at least the top 20 features whenever possible, as this allows a broader understanding of the contribution of the most important variables. Furthermore, future studies should report all model features used in the ML analysis, not only the top-ranked ones, to ensure full transparency and allow readers to verify which HL components were excluded or had low impact in the model. Based on SHAP values, we propose the following XAI visualization techniques [94,154]:

#### Beeswarm Plot

This is the most common summary plot, where features are ranked on the y-axis from most to least contribution by their mean absolute SHAP value. The x-axis represents the SHAP values, which express the change in the log-odds (or model output), resulting in a positive or negative contribution for a specific observation. Each dot represents an observation in the dataset, and the color is indicative of the original value for that observation. Higher values are displayed as red and lower values as blue. The vertical dotted line represents the zero SHAP contribution. Contributions to the right assign a positive effect, and those to the left assign a negative effect.

#### Dependency Plots

Scatter plots that show the effect a single feature has on the model’s predictions. Each point represents an instance’s feature value and its corresponding SHAP value. This plot provides a detailed and clear explanation of the direction and magnitude of the relationship (whether linear or nonlinear) between the feature and the outcome.

#### Bar Plot

This plot provides information on the global feature importance. Features are ordered on the y-axis from the highest to the lowest average contribution to the outcome.

#### Waterfall Plot

This plot is a local explanation that illustrates the contribution of each feature in transforming the expected value E[f(x)] into the final prediction f(x) for a specific instance. Each row represents a feature’s contribution; positive contributions are red, while negative contributions are blue.

#### Force Plot

This plot is a local explanation that shows the effect and the direction of the most impactful features for a given observation.

### Software

Python and R were the most widely used software in the review. However, the variety of libraries can complicate the process. To address this, the metapackage “tidymodels” provides unified syntax, enhancing replicability [135]. For DL, Python offers a more powerful environment thanks to PyTorch and TensorFlow frameworks and the possibility of developing analyses on cloud services such as Google Colab. Furthermore, we encourage researchers to make their code publicly available in open repositories such as GitHub.

### Limitations

At the level of the review itself, some limitations emerge. Although time-series data were frequently used in the analysis of lifestyle behaviors, the primary focus of this review was on the methodological framework of supervised ML, rather than on specific time-series modeling approaches. Future research should specifically address this gap by examining the current use of time-series models applied to wearable data to better capture human behavior. Moreover, the exclusion of UL algorithms limits the scope of ML algorithms covered in this review. For example, cluster analysis has been used to classify children according to their eating behaviors and identify features related to obesity [155], with findings suggesting that interventions such as reducing eating speed may help prevent childhood obesity. This scoping review focuses on supervised ML approaches, but future research could examine the current application of UL in lifestyle data. Finally, a further limitation of this review is that the search strategy prioritized studies using the umbrella term “healthy lifestyle.” While this approach captured a representative sample of research explicitly conceptualized as HL, it may have excluded multidimensional studies examining combinations of physical activity, diet, sleep, or stress that did not use this terminology. Although an interaction block was incorporated to address the interconnectedness among these domains, we acknowledge that some multidomain research may not have been retrieved due to the specificity of the search string.

### Conclusion

This review has identified several limitations within the studies reviewed that need to be addressed. First, ensuring data quality remains a significant challenge that must be addressed by carefully selecting data acquisition methods to build reliable and robust models. Second, the evaluation process is crucial for preventing overfitting, and using hold-out cross-validation can lead to high variance partitioning. Therefore, it is recommended to implement k-fold cross-validation at various stages, such as during validation; for time-series data, rolling forecast origin resampling is recommended [129].

In conclusion, this scoping review provides a comprehensive analysis of lifestyle using ML models and serves as a guideline for future research. While the relationship between lifestyle and health is well-established, ongoing efforts are needed to refine how we measure lifestyle to create robust models. It is essential to focus not only on model performance but also on data representativeness, which is closely related to the granularity established during data collection. Although RF algorithms are prominent in lifestyle data analysis, it is recommended to compare their performance with other algorithms within and across families. Future research should also incorporate SHAP values to enhance interpretability within the ML workflow. Additionally, the *tidymodels* metapackage (R software) with H2O for XAI can assist researchers in evaluating process quality with unified syntax, thereby contributing to replicability.

## Supplementary material

10.2196/78648 Multimedia Appendix 1 Extended data and detailed analyses of the included studies.

10.2196/78648 Checklist 1 PRISMA checklist.

10.2196/78648 Checklist 2 Reporting checklist for machine learning analysis in healthy lifestyle data.

## Abbrevations

AI: artificial intelligence
DASS: Depression Anxiety Stress Scale
FFQ: Food Frequency Questionnaire
FNN: fuzzy neural network
GPAQ: Global Physical Activity Questionnaire
HL: healthy lifestyle
IMS-PAQ: Indian Migration Study Physical Activity Questionnaire
INPLASY: International Platform of Registered Systematic Review and Meta-Analysis Protocols
IPAQ: International Physical Activity Questionnaire
LIME: local interpretable model-agnostic explanations
ML: machine learning
NN: neural network
NutSo-HH: Nutritional and Social Healthy Habits Scale
PRESS: Peer Review of Electronic Search Strategies
PRISMA-S: Preferred Reporting Items for Systematic Reviews and Meta-Analyses–Search extension
PRISMA-ScR: Preferred Reporting Items for Systematic Reviews and Meta-Analyses extension for Scoping Reviews
RF: random forest
SHAP: Shapley Additive Explanation
SL: supervised learning
SMOTE: synthetic minority oversampling technique
SQUASH: Short Questionnaire to Assess Health-Enhancing Physical Activity
SVM: support vector machine
UL: unsupervised learning
XAI: explainable artificial intelligence
