# Supplementary material for: Machine Learning for the Analysis of Healthy Lifestyle Data: Scoping Review and Guidelines
Source: JMIR Hum Factors. 2026 Feb 27;13:e78648. doi: 10.2196/78648 (PMC12954701; doi:10.2196/78648)
Supplement: Multimedia Appendix 1 [file humanfactors-v13-e78648-s001.docx]

**Table S1**. Search strategy.

| Database | Syntax |
| --- | --- |
| PubMed | ((("Healthy Lifestyle"[Mesh] OR "health lifestyle*"[Title/Abstract] OR "healthy lifestyle*"[Title/Abstract] OR "Health Behavior"[Mesh] OR "health behavio*"[Title/Abstract] OR "healthy behavio*"[Title/Abstract] OR  (("Sleep Hygiene"[Mesh] OR "sleep hygiene"[Title/Abstract] OR "hygiene, sleep"[Title/Abstract] OR "good sleep habit*"[Title/Abstract] OR "Sleep Quality"[Mesh] OR "sleep qualit*"[Title/Abstract] OR "sleep quant*"[Title/Abstract])  AND  ("Sedentary Behavior"[Mesh] OR "sedentary behavio*"[Title/Abstract] OR "behavior, sedentary"[Title/Abstract] OR "sedentary lifestyle"[Title/Abstract] OR "physical inactivity"[Title/Abstract] OR "inactivity, physical"[Title/Abstract] OR "lack of physical activity"[Title/Abstract] OR "Exercise"[Mesh] OR "exercise*"[Title/Abstract] OR "physical activit*"[Title/Abstract])  AND  ("Diet, Healthy"[Mesh] OR "diet, health*"[Title/Abstract] OR "health diet*"[Title/Abstract] OR "healthy diet*"[Title/Abstract] OR "health nutrition*"[Title/Abstract] OR "healthy nutrition*"[Title/Abstract] OR "health eat*"[Title/Abstract] OR "healthy eat*"[Title/Abstract])  AND  ("Stress, Psychological"[Mesh] OR "Stress, Physiological"[Mesh] OR “stress” [Title/Abstract])))  AND  ("Machine Learning"[Mesh] OR "machine learning"[Title/Abstract] OR "learning, machine"[Title/Abstract] OR "supervised learning"[Title/Abstract] OR "unsupervised learning"[Title/Abstract] OR "transfer learning"[Title/Abstract] OR "Deep Learning"[Mesh] OR "deep learning"[Title/Abstract] OR "learning, deep"[Title/Abstract] OR "Artificial Intelligence"[Mesh] OR "Artificial Intelligence"[Title/Abstract] OR "Computational Intelligence"[Title/Abstract] OR "Machine Intelligence"[Title/Abstract] OR "Computer Vision System*"[Title/Abstract] OR "Neural Networks, Computer"[Mesh] OR "Neural Network, Computer"[Title/Abstract] OR "Neural Networks, Computer"[Title/Abstract] OR "Computer Neural Network*"[Title/Abstract] OR "Neural Network Model*"[Title/Abstract] OR "perceptron*"[Title/Abstract])) NOT ("Robot*"[Title/Abstract] OR "Reinforcement Learning"[Title/Abstract] OR "Deep Reinforcement Learning"[Title/Abstract])) NOT ("systematic review"[Title/Abstract] OR "review"[Title/Abstract] OR "meta-analys*"[Title/Abstract]) |
| PsycINFO | (((MAINSUBJECT.EXACT("Health Behavior") OR tiab("health lifestyle*") OR tiab("healthy lifestyle*") OR tiab("health life-style*") OR tiab("health behavio*") OR tiab("healthy behavio*") OR ((MAINSUBJECT.EXACT("Physical Activity") OR MAINSUBJECT.EXACT("Exercise") OR MAINSUBJECT.EXACT("Sedentary Behavior") OR tiab("exercise*") OR tiab("physical activit*") OR tiab("sedentary behavio*") OR tiab("behavior, sedentary") OR tiab("sedentary lifestyle") OR tiab("physical inactivity") OR tiab("inactivity, physical") OR tiab("lack of physical activity")) AND  (MAINSUBJECT.EXACT("Healthy Eating") OR tiab("diet, health*") OR tiab("health diet*") OR tiab("healthy diet*") OR tiab("health nutrition*") OR tiab("healthy nutrition*") OR tiab("health eat*") OR tiab("healthy eat*")) AND  (MAINSUBJECT.EXACT("Sleep Quality") OR tiab("sleep hygiene") OR tiab("hygiene, sleep") OR tiab("good sleep habit*") OR tiab("sleep qualit*") OR tiab("sleep quant*")) AND  MAINSUBJECT.EXACT("Stress")  ))AND  (MAINSUBJECT.EXACT("Machine Learning") OR MAINSUBJECT.EXACT("Machine Learning Algorithms") OR tiab("machine learning") OR tiab("learning, machine") OR tiab("supervised learning") OR tiab("unsupervised learning") OR tiab("transfer learning") OR MAINSUBJECT.EXACT("Artificial Intelligence") OR tiab("Artificial Intelligence") OR tiab("Computational Intelligence") OR tiab("Machine Intelligence") OR tiab("Computer Vision System*") OR MAINSUBJECT.EXACT("Neural Networks") OR MAINSUBJECT.EXACT("Artificial Neural Networks") OR MAINSUBJECT.EXACT("Deep Neural Networks") OR tiab("deep learning") OR tiab("learning, deep") OR tiab("deep reinforcement learning") OR tiab("Neural Network, Computer") OR tiab("Neural Networks, Computer") OR tiab("Computer Neural Network*") OR tiab("Neural Network Model*") OR tiab("perceptron*")))  NOT  tiab("Robot*" OR "Reinforcement Learning" OR "Deep Reinforcement Learning"))  NOT  tiab("systematic review" OR "review" OR "meta-analys*") |
| Web of Science | ((TS=("health lifestyle*" OR "healthy lifestyle*" OR "health behavio*" OR "healthy behavio*" OR  (("sleep hygiene" OR "hygiene, sleep" OR "good sleep habit*" OR "sleep qualit*" OR "sleep quant*") AND  ("sedentary behavio*" OR "behavior, sedentary" OR "sedentary lifestyle" OR "physical inactivity" OR "inactivity, physical" OR "lack of physical activity" OR "exercise*" OR "physical activit*") AND  ("diet, health*" OR "health diet*" OR "healthy diet*" OR "health nutrition*" OR "healthy nutrition*" OR "health eat*" OR "healthy eat*") AND  “stress”))  AND  TS=("machine learning" OR "learning, machine" OR "supervised learning" OR "unsupervised learning" OR "transfer learning" OR "deep learning" OR "learning, deep" OR "Artificial Intelligence" OR "Computational Intelligence" OR "Machine Intelligence" OR "Computer Vision System*" OR "Neural Network, Computer" OR "Neural Networks, Computer" OR "Computer Neural Network*" OR "Neural Network Model*" OR "perceptron*")) NOT TS=("Robot*" OR "Reinforcement Learning" OR "Deep Reinforcement Learning")) NOT TS=("systematic review" OR "review" OR "meta-analys*") |

**Table S2**. Demographic characteristics, sample size, and data source of the included studies.

| Study | Continent (Country) | Sample size (% females) | Data source |
| --- | --- | --- | --- |
|  |  |  |  |
| Abdul Rahman et al., 2023 [1] | Asia (Brunei Darussalam, Indonesia, Malaysia, Philippines, Singapore, Thailand, and Vietnam) | 15366 (53%) | self-acquired |
| Afrash et al., 2022 [2] | Asia (Iran) | 3930 (100%) | self-acquired |
| Ai et al., 2023 [3] | America (Canada) | 139 (70%) | Private |
| Allen, 2023 [4] | America (United States of America) | 3142 (no reported) | Public |
| Alshuraf et al., 2017 [5] | America (United States of America) | 39 (100%) | self-acquired |
| Birk et al., 2021[6] | Asia (India) | 5655 (47%) | Private |
| Bôto et al., 2022 [7] | Europe (Portugal) | 325 (53%) | self-acquired |
| Butkevičiūtė et al., 2023 [8] | no reported | 98 (58%) | self-acquired |
| Cai et al., 2020 [9] | Asia (China) | 890 (67%) | self-acquired |
| Cheung et al., 2017 [10] | America (United States of America) | 79 (42%) | self-acquired |
| Chiang and Dey, 2019 [11] | America (United States of America) | 8 (50%) | self-acquired |
| Cortés-Ibañez, Nagaraj, Cornelissen, Navis, et al., 2021 [12] | Europe (Netherlands) | 8464 (61%) | Private |
| Cortés-Ibañez, Nagaraj, Cornelissen, Sidorenkov, et al., 2021 [13] | Europe (Netherlands) | 110384 (59%) | Private |
| Dianati-Nasab et al., 2024 [14] | Asia (Iran) | 2018 (100%) | self-acquired |
| Faruqui et al., 2019 [15] | America (United States of America) | 10 (no reported) | self-acquired |
| Gu et al., 2025 [16] | America (United States of America) | 873 (100%) | Public |
| Guthrie et al., 2019 [17] | no reported | 135 (83%) | self-acquired |
| Hu, Liu, & Li, 2020 [18] | America (United States of America) | 26698 (no reported) | Public |
| Hu, Liu, Ji, et al., 2020 [19] | America (United States of America) | 26697 (no reported) | Public |
| Huang et al., 2023[20] | Asia (China) | 2128 (no reported) | Public |
| Jin & Halili, 2025 [21] | Asia (China) | 8637 (58.95%) | Private |
| Kim et al., 2024 [22] | Asia (Korea) | 4048 (57%) | Private |
| Kimura et al., 2023 [23] | Asia (Japan) | 122 (56%) | self-acquired |
| Kiss et al., 2022 [24] | America (United States of America) | 11878 (50%) | Private |
| Li and Song, 2025 [25] | Asia (China) | 12241 (not reported) | Private |
| Lim et al., 2019 [26] | Asia (Korea) | 30 (41%) | Self-acquired |
| Lim et al., 2022 [27] | Asia (Korea) | 5749 (no reported) | Private |
| Lin et al., 2024 [28] | Asia (China) | 15874 (no reported) | Private |
| Liu et al., 2025 [29] | Asia (China) | 9271 (46.76%) | Private |
| Luo et al., 2025 [30] | Europe (United Kingdom) | 470778 (50%) | Private |
| Luo et al., 2022 [31] | Asia (China) | 12541 (no reported) | self-acquired |
| Luo et al., 2025 [32] | America, Europe, Asia (United States of America, United Kingdom, China) | 14581 (no reported) | Private |
| Majcherek et al., 2022 [33] | Europe (30 countries) | 140791 (no reported) | Public |
| Majcherek et al., 2025 [34] | America (United States of America) | 253680 (56%) | Public |
| Matta et al., 2018 [35] | Asia (Lebanon) | 31 (61%) | self-acquired |
| Moon and Woo, 2024 [36] | Asia (Korea) | 213820 (38.2%) | self-acquired |
| Morris et al., 2023 [37] | America (United States of America) | 3980 (64%) | self-acquired |
| Mousavi et al., 2022 [38] | Asia (Iran) | 1428 (73%) | self-acquired |
| Mun and Geng, 2019 [39] | America (United States of America) | 212 (52%) | Self-acquired |
| Nichols et al., 2022 [40] | America, Europe, Asia (United States of America, United Kingdom, Canada, India) | 804 (100%) | Self-acquired |
| Oladeji et al., 2021 [41] | Africa (52 African countries) | no reported | Public |
| Park et al., 2024 [42] | America (United States of America) | 1764 (53%) | Self-acquired |
| Park & Edington, 2004 [43] | Asia (Korea) | 425148 (no reported) | Private |
| Park, 2024 [44] | Asia (Korea) | 65611 (66%) | Private |
| Pereira et al., 2025 [45] | Europe (Portugal) | 274 (89.4%) | Self-acquired |
| Puterman et al., 2020 [46] | America (United States of America) | 13611 (59%) | Public |
| Qasrawi et al., 2023 [47] | Asia (Palestine) | 6373 (50%) | Public |
| Recenti, Ricciardi, Edmunds, Gislason, et al., 2021 [48] | Europe (Iceland) | 2943 (no reported) | self-acquired |
| (Recenti, Ricciardi, Edmunds, Jacob, et al., 2021 [49] | Europe (Iceland) | 3137 (no reported) | self-acquired |
| Ren et al., 2025 [50] | Asia (China) | 2688 (46.76%) | Private |
| Ruiz et al., 2024 [51] | America (Colombia) | 680 (55.1%) | self-acquired |
| Sandri et al., 2025 [52] | Europe (Spain) | 22181 (80.8%) | self-acquired |
| Sathyanarayana et al., 2016 [53] | Asia (Qatar) | 322 (66%) | self-acquired |
| Shi et al., 2025 [54] | America (United States of America) | 3902 (41.33%) | Public |
| Staudenmayer et al., 2015 [55] | America (United States of America) | 20 (50%) | self-acquired |
| Stemmer et al., 2022 [56] | no reported | 661 (no reported) | Public |
| Su et al., 2025 [57] | Asia (China) | 843 (66.9%) | self-acquired |
| Wallace et al., 2019 [58] | America (United States of America) | 8668 (54%) | Public |
| Wallace et al., 2021 [59] | America (United States of America) | 6749 (52%) | Public |
| Wang et al., 2025 [60] | Asia (China) | 471 (54.1%) | self-acquired |
| Xin & Ren, 2022 [61] | Asia (China) | 1460 (58%) | Public |
| Zhang et al., 2024 [62] | Asia (China) | 1882 (55%) | self-acquired |
| Zhou et al., 2019 [63] | America (United States of America) | 210 (100%) | self-acquired |
| Zhou et al., 2022 [64] | America (United States of America) | 3019 (no reported) | Public |
| Zhou et al., 2025 [65] | Europe (United Kingdom) | 186812 (52%) | Private |

**Figure S1**. Geographical distribution of the included studies. Sample size is represented as a continuous variable, where larger circles represent larger samples. Data sources are depicted using colors: private datasets are colored in red, public in blue, and self-acquired data in green.


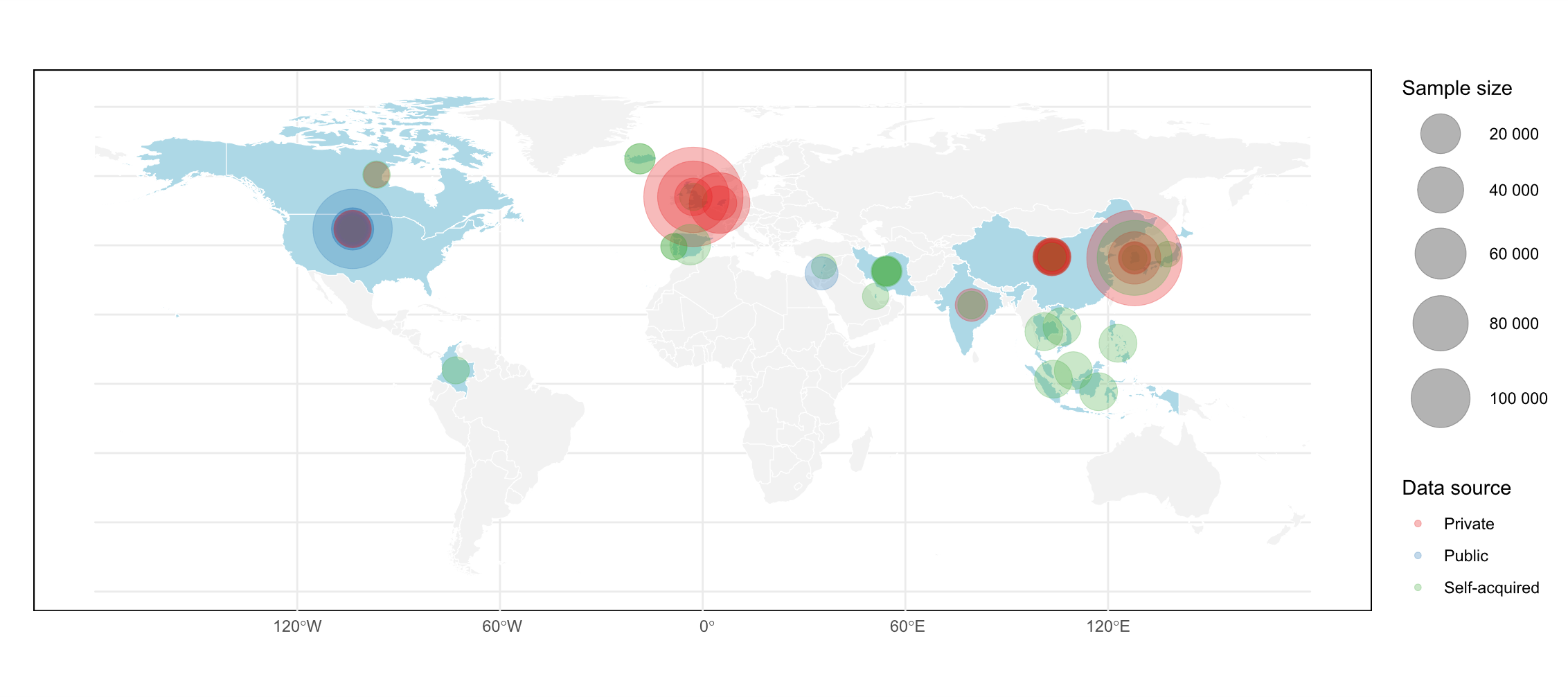


**Table S3**. Data acquisition methodology for each lifestyle component and health outcomes across the included studies. Questionnaire (Standarized or not). Single Item (Category). Sensor (Type of sensor). Words (Source).

| Study | Physical Activity | | Diet | | Sleep | | Stress | | Health outcome/s |
| --- | --- | --- | --- | --- | --- | --- | --- | --- | --- |
|  | Methodology | Description | Methodology | Description | Methodology | Description | Methodology | Description |  |
| Abdul Rahman et al., 2023 [1] | Questionnaire (Standarized) and Item (Frequency) | Global Physical Activity Questionnaire (GPAQ)(physical activity levels (METs). Sedentary hours. | Single items (Frequency) | High sugar beverage, Salt intake, fruits/vegetables intake | Single items (Sleep hours) | Sleep hours |  |  | Mental health |
| Afrash et al., 2022 [2] | Single items (Engagement) | Regular physical activity (yes/no) | Single items (Frequency) | Dairy products status (rarely, sometimes, highly) |  |  |  |  | Cancer |
| Ai et al., 2023 [3] | Questionnaire (Standarized) and Item (Frequency) | The lifetime total physical activity questionnaire (Exercise and Sport sub-sacale) | Questionnaire (non Standarized) | The diet habit questionnaire (son 4 items de un quest y 22 de otro) |  |  | Questionnaire (Standarized) | Stress subscale from the depression anxiety stress (DASS) | Alzheimer |
| Allen, 2023 [4] | Single items (Environment) | Physical inactivity, Access to exercise opportunities | Single items (Environment) | Food environment index, Food insecurity, Limited access to healthy foods | Single items (Sleep hours) | Insufficient sleep |  |  | Obesity |
| Alshuraf et al., 2017 [5] | Sensor (Wearable) | total activity level and time in low, medium and high activity | Single items (Habits) | Nutrition questions |  |  | Questionnaire (Standarized) | INTERHEART STRESS | Cardiovascular disease |
| Birk et al., 2021 [6] | Questionnaire (Standarized) | Indian Migration Study Physical Activity Questionnaire (IMS-PAQ) | Questionnaire (Standarized) | Food Frequency Questionnaire (FFQ) |  |  |  |  | Diabetes |
| Bôto et al., 2022 [7] | Single items (Engagement) | Usual forms of activity | Single items (Habits) | dietary habits | Single items (Sleep hours) | Usual time of waking up, usual time of going to bed |  |  | Lifestyle (Diet) |
| Butkevičiūtė et al., 2023 [8] | Sensor (Wearable) | chest band (physical conditioning test) |  |  |  |  |  |  | Lifestyle (Physical activity) |
| Cai et al., 2020 [9] | Questionnaire (Standarized) | Physical fitness test |  |  |  |  |  |  | Successful aging |
| Cheung et al., 2017 [10] | Sensor (wearable) | periods of exercise |  |  |  |  | Single items (Stress level) | stress perception, appraisal, and sources | Lifestyle (Physical activity) |
| Chiang and Dey, 2019 [11] | Sensor (wearable) | calories burned, steps, distance, floors climbed, sedentary minutes, lightly active minutes, fairly active minutes, very active minutes, exercise calories |  |  | Sensor (wearable) | minutes asleep, minutes awake, awekening times, bedtime, wake up time |  |  | Blood Pressure |
| Cortés-Ibañez, Nagaraj, Cornelissen, Navis, et al., 2021 [12] | Questionnaire (Standarized) and Item (Frequency) | Short Questionnaire to Assess Health-enhancing Physical Activity (SQUASH), sedentary behavior (number of hours watching tv per day) | Questionnaire (Standarized) | Food Frequency Questionnaire (FFQ) |  |  |  |  | Cancer |
| Cortés-Ibañez, Nagaraj, Cornelissen, Sidorenkov, et al., 2021 [13] | Questionnaire (Standarized) and Item (Frequency) | Short Questionnaire to Assess Health-enhancing Physical Activity (SQUASH), sedentary behavior (number of hours watching tv per day) | Questionnaire (Standarized) | Food Frequency Questionnaire (FFQ) |  |  |  |  | Cancer |
| Dianati-Nasab et al., 2024 [14] | Single items (Engagement) | Physical activity (no/yes) |  |  |  |  |  |  | Cancer |
| Faruqui et al., 2019 [15] | Single items (Frequency) | Activity time | Single items (Type of products) | Daily diet information, where collected food intake data (breakfast, lunch, dinner, snacks) is discretized into calories, macronutrient content (carbohydrates and fat) |  |  |  |  | Diabetes |
| Gu et al., 2025 [16] | Single items (Frequency) | Self-reported physical activity frequency | Single items (Frequency) | Daily intake | Single items (Sleep hours) | sleep duration |  |  | Infertility risk in women |
| Guthrie et al., 2019 [17] | Single items (Frequency) | Minutes of physical activity, physical activity reported | Single items (Type of products) | (meals reported, plant-based meals reported) |  |  |  |  | Cardiometabolic disease |
| Hu, Liu, & Li, 2020 [18] | Single items (Frequency) | No leisure-time physical activity |  |  | Single items (Sleep hours) | insufficient sleep |  |  | Cardiovascular disease |
| Hu, Liu, Ji, et al., 2020 [19] | Single items (Frequency) | No leisure-time physical activity |  |  | Single items (Sleep hours) | Sleeping <7 h |  |  | Cardiovascular disease |
| Huang et al., 2023 [20] | Single items (Engagement) | Exerciseing (yes/no) |  |  | Single items (Sleep quality) | Sleep quality (Very good, Good, Fair, Poor, Very Poor) |  |  | Cognitive impairment |
| Jin & Halili, 2025 [21] | Single items (Intensity) | Low, Moderate, High |  |  | Single items (Sleep hours) | sleep duration |  |  | Depression risk |
| Kim et al., 2024 [22] | Questionnaire (Standarized) | Korean global physical activity questionnaire, metabolic equivalent task (MET) | Questionnaire (Standarized) | Food Frequency Questionnaire (FFQ) | Questionnaire (Standarized) and Single items (Sleep hours) | Pittsburg Sleep Quality Index (PSQI), sleep duration | Questionnaire (Standarized) | Psychosocial Well-being Index-Short Form | Quality of life |
| Kimura et al., 2023 [23] | Sensor (Wearable) | Metabolic equivalent task (MET), heart rate, exercise habits, exercise frequency |  |  | Sensor (Wearable) | total sleep time, sleep onset, sleep eficiency, awaking time, nap time |  |  | Alzheimer |
| Kiss et al., 2022 [24] | Single items (Engagement) | outdoor activities |  |  | Questionnaire (Standarized) and Single items (Sleep hours) | sleep timing, duration, sleep onset latency, sleep inertia (time taken to get out of bed) on school and free days, and chronotype-proxy. Munich Chronotype questionnaire and Sleep Disturbance Scale for Children | Questionnaire (Standarized) | The 4-item Perceived Stress Scale | Mental health |
| Li & Song, 2025 [25] | Single items (Engagement) | Sports social capita index (social activities, trust, sports support and physical activity) |  |  |  |  |  |  | Cognitive ability |
| Lim et al., 2019 [26] | Single items (Intensity) | Exercise intensity, activity index |  |  | Questionnaire (Standarized) and Single items (Sleep quality) | Pittsburg Sleep Quality Index (PSQI), subjective sleep quality, sleep disturbance, dream issues, feeling after sleep, body condition after sleep | Single items (Stress level) | stress level | Lifestyle (Sleep) |
| Lim et al., 2022 [27] | Single items (Intensity) | Physical activity was defined as over 10 min of medium-strength vigorous physical activity, which involved breathing slightly fast or faster than usual. |  |  |  |  |  |  | Osteoarthritis |
| Lin et al., 2024 [28] | Single items (Engagement) | Regular physical activity (yes/no) |  |  |  |  |  |  | Feeling Lonely |
| Liu et al., 2025 [29] | Questionnaire (Standarized) | Global Physical Activity Questionnaire (GPAQ) |  |  |  |  |  |  | Cardiovascular mortality |
| Luo et al., 2025 [30] | Single items (Intensity) | Activities, metabolic equivalent of task (MET) | Questionnaire (Standarized) | Food Frequency Questionnaire (FFQ) | Single items (Sleep hours) | An adequate sleep was defined as having a sleep duration of 7–9 h/day | Single items (Stress level) | stress in the past 2 years | Chronic kidney disease |
| Luo et al., 2022 [31] | Single items (Frequency) | Self-reported physical activity frequency |  |  | Single items (Sleep hours) | sleep duration | Single items (Stress level) | Academic stress | Social network adiction risk |
| Luo et al., 2025 [32] | Single items (Intensity) | Low, Moderate, High | Single items (Habits) | Consume fruit daily, consume vegetables daily | Single items (Sleep hours) | sleep duration |  |  | Fraility index |
| Majcherek et al., 2022 [33] | Single items (Frequency) | time spent doing sports, fitness or recreational (leisure) physical activities in a typical week (in minutes), time spent walking to get to and from places on a typical day | Single items (Type of products) | fruits portion: the number of portions of fruit a day, excluding juice. Vegetables portion: number of portions of vegetables or salad a day, excluding juice and potatoes | Single items (Sleep problems) | Sleeping problems: having trouble falling or staying asleep or sleeping too much over the previous two weeks |  |  | Mental health |
| Majcherek et al., 2025 [34] | Single items (Engagement) | Regular physical activity | Single items (Habits) | Consume fruit daily, consume vegetables daily |  |  |  |  | Diabetes risk |
| Matta et al., 2018 [35] | Sensor (wearable) | heart rate variability |  |  |  |  |  |  | Lifestyle (Sleep) |
| Moon & Woo, 2024 [36] |  |  | Single items (Habits) | frequency of sugary drink intake, consumption of fast food, fruit intake, breakfast intake, and water intake | Single items (Sleep quality) | sleep quality, fatigue recovery from sleep | Single items (Stress level) | stress level | General anxiety disorder |
| Morris et al., 2023 [37] | Questionnaire (Standarized) and Single items (Environment) | Physical Activity scale from the Active Living Index, environmental data reflecting densities of physical activity resources | Questionnaire (Standarized) and Single items (Environment) | Food Frequency Questionnaire (FFQ), environmental data reflecting densities of “favorable” food stores |  |  | Questionnaire (Standarized) | Global Perceived Stress Scale | Cardiovascular disease |
| Mousavi et al., 2022 [38] | Single items (Engagement) | Exercise (yes/no) | Single items (Frequency) | Most consumed meal, Having breakfast, meals time, eating speed | Single items (Sleep hours) | Sleep time |  |  | Lifestyle (Diet) |
| Mun & Geng, 2019 [39] | Single items (Frequency) | Question: “How often do you do light or moderate leisure-time physical activities for at least 10 minutes that cause only light sweating or a slight moderate increase in breathing or heart rate? |  |  | Single items (Sleep hours) | Question: how many hours of sleep did you get last night? | Questionnaire (Standarized) and Sensor (Wearable) | hear rate variability, profile of mood states POMS | Fatigue |
| Nichols et al., 2022 [40] | Questionnaire (Standarized) | Pregnancy physical activity questionnaire | Questionnaire (Standarized) | PrimeScreen questionnaire | Questionnaire (Standarized) | Pittsburg Sleep Quality Index (PSQI) | Questionnaire (Standarized) | perceived stress scale (PSS) | Lifestyle (Stress) |
| Oladeji et al., 2021 [41] | Words | terms about exercise | Words | terms about diet |  |  |  |  | Obesity |
| Park et al., 2024 [42] | Single items (Intensity) | metabolic equivalent task (MET) |  |  |  |  |  |  | Adverse health event |
| Park & Edington, 2004 [43] | Single items (Frequency) | exercise (regular activity) | Single items (Type of products) | diet in fiber and in fatty food | Single items (Sleep hours) | hours of sleep | Single items (Stress level) | Appraised stress score | Diabetes |
| Park, 2024 [44] | Single items (Frequency) | Regular exercise undertaken | Questionnaire (Standarized) | Food Frequency Questionnaire (FFQ) |  |  |  |  | Visceral fat |
| Pereira et al., 2025 [45] | Questionnaire (Standarized) | International Physical Activity Questionnaire (IPAQ) | Questionnaire (Standarized) | Food Frequency Questionnaire | Questionnaire (Standarized) | Pittsburg Sleep Quality Index (PSQI) | Questionnaire (Standarized) | Stress subscale from the depression anxiety stress (DASS) | Bournout risk |
| Puterman et al., 2020 [46] | Single items (Intensity) | Low/no vigorous activity |  |  | Single items (Sleep problems) | sleep problems |  |  | Mortality |
| Qasrawi et al., 2023[47] | Single items (Intensity) | Low, Moderate, High | Single items (Habits) | Healthy food consumption (yes/no) | Single items (Sleep hours) | (≥8 h per day, <8 h per day |  |  | Mental health |
| Recenti, Ricciardi, Edmunds, Gislason, et al., 2021 [48] | Single items (Frequency) | Self-reported physical activity frequency |  |  |  |  |  |  | Diabetes |
| Recenti, Ricciardi, Edmunds, Jacob, et al., 2021 [49] | Single items (Frequency) | Self-reported physical activity frequency |  |  |  |  |  |  | Lifestyle (Physical activity) |
| Ren et al., 2025[50] | Single items (Engagement) | Regular physical activity (yes/no) |  |  |  |  |  |  | Cognitive ability |
| Ruiz et al., 2024 [51] | Single items (Engagement) | Regular physical activity |  |  |  |  |  |  | Depression |
| Sandri et al., 2025 [52] | Questionnaire (Standarized) | Nutritional and Social Healthy Habits Scale (NutSo-HH) | Questionnaire (Standarized) | Nutritional and Social Healthy Habits Scale (NutSo-HH) | Questionnaire (Standarized) | Nutritional and Social Healthy Habits Scale (NutSo-HH) |  |  | Lifestyle (Diet) |
| Sathyanarayana et al., 2016 [53] | Sensor (wearable) | actigraphy |  |  | Sensor (wearable) | actigraphy |  |  | Lifestyle (Sleep) |
| Shi et al., 2025 [54] | Single items (Frequency) | Self-reported physical activity frequency | Single items (Frequency) | Daily intake | Single items (Sleep hours) | sleep duration |  |  | Osteoporosis |
| Staudenmayer et al., 2015 [55] | Sensor (wearable) | actigraphy |  |  |  |  |  |  | Lifestyle (Physical activity) |
| Stemmer et al., 2022[56] | Words | Keywords related to fitness | Words | Keywords related to nutrition |  |  |  |  | Inflammatory bowel disease |
| Su et al., 2025 [57] |  |  |  |  | Questionnaire (Standarized) | Pittsburg Sleep Quality Index (PSQI) |  |  | Resilience |
| Wallace et al., 2019 [58] | Questionnaire (Standarized) | physical activity scale for the elderly |  |  | Questionnaire (Standarized) and Single items (Sleep hours) | Total sleep time, bed time, wake-up time, time in bed, sleep efficiency, sleep latency, napping. Self-reported symptoms of sleep disorders. Medication with effects on sleep. Epworth Sleepiness Scale. Pittsburgh Sleep Quality Index |  |  | Mortality |
| Wallace et al., 2021 [59] |  |  |  |  | Sensor (Polysomnography) and Single items (Sleep hours) | Polysomnography, self-report sleep (Frequency of difficulty getting back to sleep, Frequency of excessive daytime sleepiness, Sleep duration) |  |  | Mortality |
| Wang et al., 2025 [60] |  |  | Questionnaire (Standarized) | Mini Nutritional Assessment (MNA) | Questionnaire (Standarized) | Pittsburg Sleep Quality Index (PSQI) |  |  | Lifestyle (Sleep) |
| Xin and Ren, 2022 [61] | Single items (Frequency) | regular exercise |  |  | Single items (Sleep hours) | sleep duration |  |  | Mental health |
| Zhang et al., 2024 [62] | Single items (Frequency) | sports habits (frequency of engagement in physical activity per week and types of sports) | Single items (Habits) | eating habits (consumption of fatty meal, vegetables, and fruits) | Questionnaire (Standarized) | Pittsburg Sleep Quality Index (PSQI) | Single items (Stress level) | stress index | Lifestyle (Sleep) |
| Zhou et al., 2019 [63] | Sensor (Wearable) | metabolic equivalent task (MET), steps |  |  |  |  |  |  | Lifestyle (Physical activity) |
| Zhou et al., 2022 [64] | Sensor (Phone) | points of interes fitness and sports centers, and nature parks | Sensor (Phone) | (points of interes fast-food restaurants) |  |  |  |  | Obesity |
| Zhou et al., 2025 [65] | Single items (Intensity) | metabolic equivalent task (MET) | Single items (Habits) | Nutrition questions |  |  |  |  | Late-onset psoriasis risk |

**Table S4**. Preprocessing.

| Study | Variable transformation | Missing imputation | Resampling | Dimensionality reduction |
| --- | --- | --- | --- | --- |
| Abdul Rahman et al., 2023 [1] | No reported | 50% of missing values were excluded from the analysis. Missing data were handled using multiple imputation by chained equations (MICE) | Synthetic Minority Oversampling Technique (SMOTE) | Wrapper methods (stepwise selection and Boruta method) and embedded method (Random Forest: Gini Index) |
| Afrash et al., 2022 [2] | Normalization (mean = 0, var = 1), and min-max scalar | 50% of missing values were excluded from the analysis. Single missing imputation with mean and mode | No reported | Wrapper methods (Genetic Algorithm) |
| Ai et al., 2023 [3] | Z-score normalization, and statistical transformation | Remove missing values | No reported | Filter methods (No reported) |
| Allen, 2023[4] | No reported | 10% of missing values were excluded from the analysis. | No reported | Filter methods (correlation) |
| Alshuraf et al., 2017 [5] | Statistical transformation | Single missing imputation with mean | No reported | Filter and wrapper methods (correlation, information gain, gain ratio, Chi-square, principal component analysis) |
| Birk et al., 2021 [6] | No reported | No reported | Synthetic Minority Oversampling Technique (SMOTE) | Embedded methods (LASSO, ElasticNet and Random Forest) |
| Bôto et al., 2022 [7] | Logaritmic transformation | No reported | No reported | Embedded methods (Decision Tree) |
| Butkevičiūtė et al., 2023 [8] | Standarization | No reported | No reported | No reported |
| Cai et al., 2020 [9] | Range method | No reported | No reported | Embedded methods (Random Forest: Gini index; Deep Learning: Gordon method) |
| Cheung et al., 2017 [10] | No reported | No reported | No reported | Embedded methods (Random Forest) |
| Chiang and Dey, 2019 [11] | Normalization (mean = 0 variance = 1), and categorical variables were one-hot encoded | Remove missing values in the output. Missings imputation with K Nearest Neighbor (KNN) K=5 | No reported | Embedded methods (Random Forest: bagging) |
| Cortés-Ibañez, Nagaraj, Cornelissen, Navis, et al., 2021 [12] | Uniform mean and sd | 30% of missing values were excluded from the analysis. Missing data were handled using multiple imputation by chained equations (MICE) | Sample-size equalization matching cases and controls by sex, age, and education level, and randomly selection of participants resulting in 50% cases and 50 % controls | Embedded methods (Random Forest: Gini index; ElasticNet) |
| Cortés-Ibañez, Nagaraj, Cornelissen, Sidorenkov, et al., 2021 [13] | Uniform mean and sd | No reported | Sample-size equalization method that involved randomly grouping participants with no history of cancer into 39 equal subsets based on the number of cancer survivors (107,624 cancer-free participants/2760 cancer survivors = 39 subsets) | Embedded methods (Random Forest: Gini index) |
| Dianati-Nasab et al., 2024 [14] | No reported | No reported | No reported | No reported |
| Faruqui et al., 2019 [15] | min-max scalar | Replace the missing value with the last available data (last observation) | No reported | No reported |
| Gu et al., 2025 [16] | No reported | No reported | Synthetic Minority Oversampling Technique (SMOTE) and undersampling | Wrapper method (Boruta method) |
| Guthrie et al., 2019 [17] | No reported | No reported | No reported | No reported |
| Hu, Liu, and Li, 2020 [18] | No reported | Remove missing values | No reported | Embedded methods (BART-Machine) |
| Hu, Liu, Ji, et al., 2020 [19] | No reported | Remove missing values | No reported | Embedded methods (Random Forest: Gini index; BART-Machine) |
| Huang et al., 2023 [20] | No reported | Missing data were handled using multiple imputation by chained equations (MICE) | Synthetic Minority Oversampling Technique (SMOTE) | No reported |
| Jin and Halili, 2025 [21] | Z-score, Categorical variables were one-hot-encoded | MissForest algorithm | Synthetic Minority Oversampling Technique (SMOTE) | Embedded methods (LASSO, ElasticNet) and Wrapper methods (Boruta method) |
| Kim et al., 2024 [22] | Min-max scalar | No reported | Synthetic Minority Oversampling Technique (SMOTE) | No reported |
| Kimura et al., 2023 [23] | No reported | Missing imputation with training data | No reported | Filter and wrapper methods (correlation and Boruta method) |
| Kiss et al., 2022 [24] | T-scores, and categorical variables were one hot encoded | Remove missing values | No reported | Filter methods (correlation) |
| Li and Song, 2025 [25] | Min-max scalar | No reported | No reported | No reported |
| Lim et al., 2019 [26] | Categorical variables were one-hot-encoded | Remove missing values | No reported | Embedded methods(Decision Tree and Random Forest), and other methods (Factor analysis) |
| Lim et al., 2022 [27] | Categorical recodification by PCA with quantile transformer scaler | Remove missing values | Minority class | Other methods (Principal Component Analysis ) |
| Lin et al., 2024 [28] | Min-max scalar | mean and mode | No reported | Wrapper methods (Logistic Regression) |
| Liu et al., 2025 [29] | Z-score, Categorical variables were one-hot-encoded | No reported | No reported | No reported |
| Luo et al., 2025 [30] | No reported | Missing data were handled using multiple imputation by chained equations (MICE) and regression-based algorithm. | No reported | Embedded methods (mean decrease impurity) |
| Luo et al., 2022 [31] | Normalization (mean = 0 variance = 1) | No reported | No reported | Embedded methods (Random Forest: Gini index) |
| Luo et al., 2025 [32] |  | MissForest algorithm | No reported |  |
| Majcherek et al., 2022 [33] | No reported | Remove missing values | No reported | No reported |
| Majcherek et al., 2025 [34] | No reported | No reported | Synthetic Minority Oversampling Technique (SMOTE), ADASYN | No reported |
| Matta et al., 2018 [35] | Z-score normalization, and statistical transformation | No reported | No reported | No reported |
| Moon and Woo, 2024 [36] | Z-score | K Nearest Neighbor (KNN) | Synthetic Minority Oversampling Technique (SMOTE) | Embedded methods (LASSO, SelectKBest, XGBoost) |
| Morris et al., 2023 [37] | No reported | Single missing imputation with median | No reported | No reported |
| Mousavi et al., 2022 [38] | No reported | No reported | No reported | Wrapper methods (Genetic Algorithm) |
| Mun and Geng, 2019 [39] | Standarization | Missing imputation with regression-based algorithm | No reported | Embedded methods (Random Forest: Gini index) |
| Nichols et al., 2022 [40] | Categorical recodification | Remove missing values | Minority class | Filter methods (mutual information) |
| Oladeji et al., 2021 [41] | No reported | No reported | No reported | Filter methods (correlation and near zero or zero variance) |
| Park et al., 2024 [42] | No reported | Imputation comparing to the peers in a similar health profile group | Bootstrapping | No reported |
| Park and Edington, 2004 [43] | No reported | No reported | No reported | Embedded methods (XGBoost) |
| Park, 2024 [44] | Z-score | Single missing imputation with mean and mode | No reported | Filter methods (correlation) |
| Pereira et al., 2025 [45] | Normalization (mean = 0 variance = 1) | Mean | No reported | No reported |
| Puterman et al., 2020 [46] | Standarization, and categorical recodification | Missing imputation with random forest multiple imputation | No reported | Other methods (Principal Component Analysis ) |
| Qasrawi et al., 2023 [47] | No reported | Single missing imputation with median | No reported | Filter methods (correlation) |
| Recenti, Ricciardi, Edmunds, Gislason, et al., 2021 [48] | No reported | Remove missing values | Synthetic Minority Oversampling Technique (SMOTE) | No reported |
| Recenti, Ricciardi, Edmunds, Jacob, et al., 2021 [49] | No reported | Remove missing values | Synthetic Minority Oversampling Technique (SMOTE) | No reported |
| Ren et al., 2025 [50] | No reported | Mean | Synthetic Minority Oversampling Technique (SMOTE) | No reported |
| Ruiz et al., 2024 [51] | No reported | No reported | No reported | No reported |
| Sandri et al., 2025 [52] | Z-score |  | Synthetic Minority Oversampling Technique (SMOTE) |  |
| Sathyanarayana et al., 2016 [53] | No reported | Remove missing values | No reported | No reported |
| Shi et al., 2025 [54] | Min-max scalar | Random Forest | Synthetic Minority Oversampling Technique (SMOTE) | Embedded methods (LightGBM), Filter methods (correlation) |
| Staudenmayer et al., 2015 [55] | Statistical transformation | No reported | No reported | No reported |
| Stemmer et al., 2022 [56] | No reported | No reported | No reported | No reported |
| Su et al., 2025 [57] | No reported | No reported | No reported | Embedded methods (LASSO) |
| Wallace et al., 2019 [58] | No reported | No reported | No reported | No reported |
| Wallace et al., 2021 [59] | No reported | Missing imputation with random forest multiple imputation | No reported | Filter methods (correlation) |
| Wang et al., 2025 [60] | No reported | Multiple imputation | Synthetic Minority Oversampling Technique (SMOTE) | Embedded methods (LASSO) and Wrapper methods (Boruta method) |
| Xin and Ren, 2022 [61] | No reported | No reported | No reported | Filter methods (hypothesis testing: Chi-squared and T-test) |
| Zhang et al., 2024 [62] | No reported | No reported | Synthetic Minority Oversampling Technique (SMOTE) | No reported |
| Zhou et al., 2019 [63] | No reported | No reported | No reported | No reported |
| Zhou et al., 2022 [64] | No reported | Remove missing values | No reported | Filter methods (multicollinearity) |
| Zhou et al., 2025 [65] | No reported | No reported | Synthetic Minority Oversampling Technique (SMOTE) | No reported |

**Table S5**. Evaluation metrics

| Study | Evaluation metrics |
| --- | --- |
| Abdul Rahman et al., 2023 [1] | Accuracy, specificity, sensitivity, AUC ROC, error rate, Kappa |
| Afrash et al., 2022 [2] | Accuracy, specificity, sensitivity, AUC ROC, error rate, Kappa, time |
| Ai et al., 2023 [3] | Accuracy, specificity, sensitivity, AUC ROC |
| Allen, 2023 [4] | MAE |
| Alshuraf et al., 2017 [5] | Accuracy, AUC ROC, F1-score |
| Birk et al., 2021 [6] | Specificity, sensitivity, AUC ROC |
| Bôto et al., 2022 [7] | Not reported |
| Butkevičiūtė et al., 2023 [8] | Accuracy, precision, recall, F1 score |
| Cai et al., 2020 [9] | Accuracy, specificity, sensitivity, precision, AUC ROC, F1-score |
| Cheung et al., 2017 [10] | Error rate |
| Chiang & Dey, 2019 [11] | Mean absolute error (MAE), root mean square error (RMSE), mean absolute percentage error (MAPE) |
| Cortés-Ibañez, Nagaraj, Cornelissen, Navis, et al., 2021 [12] | AUC ROC |
| Cortés-Ibañez, Nagaraj, Cornelissen, Sidorenkov, et al., 2021 [13] | AUC ROC |
| Dianati-Nasab et al., 2024 [14] | Accuracy, specificity, sensitivity, AUC ROC, Kappa |
| Faruqui et al., 2019 [15] | Mean absolute error (MAE), mean squared error (MSE) |
| Gu et al., 2025 [16] | Accuracy, Recall, F1 score, and the Matthews Correlation Coefficient (MCC), |
| Guthrie et al., 2019 [17] | Specificity, sensitivity, AUC ROC |
| Hu, Liu, and Li, 2020 [18] | Not reported |
| Hu, Liu, Ji, et al., 2020 [19] | Root mean squared error (RMSE) |
| Huang et al., 2023 [20] | Accuracy, specificity, sensitivity, AUC ROC |
| Jin and Halili, 2025 [21] | Accuracy, precision, recall, AUC ROC, F1-score |
| Kim et al., 2024 [22] | Accuracy, specificity, sensitivity, AUC ROC, F1-score |
| Kimura et al., 2023 [23] | Specificity, sensitivity, AUC ROC, F1-score |
| Kiss et al., 2022 [24] | Not reported |
| Li and Song, 2025 [25] | Accuracy, precision, recall, AUC ROC, F1-score |
| Lim et al., 2019 [26] | Accuracy |
| Lim et al., 2022 [27] | Accuracy, specificity, sensitivity, precision, confusion matrix, AUC ROC |
| Lin et al., 2024 [28] | Accuracy, precision, recall, AUC ROC, F1-score |
| Liu et al., 2025 [29] | AUC ROC, Brier scores |
| Luo et al., 2025 [30] | Not reported |
| Luo et al., 2022 [31] | Coefficient of determination (R^2^) |
| Luo et al., 2025 [32] | Root mean squared error (RMSE), MSE, coefficient of determination (R^2^) |
| Majcherek et al., 2022 [33] | Accuracy, precision, recall, confusion matrix, F1-score |
| Majcherek et al., 2025 [34] | Accuracy, specificity, sensitivity, precision, AUC ROC, F1-score, R², MSE |
| Matta et al., 2018 [35] | Accuracy, specificity, sensitivity, precision, confusion matrix, AUC ROC, error rate |
| Moon and Woo, 2024 [36] | Accuracy, specificity, sensitivity, precision, AUC ROC |
| Morris et al., 2023 [37] | Accuracy |
| Mousavi et al., 2022 [38] | Accuracy, specificity, sensitivity |
| Mun and Geng, 2019 [39] | Coefficient of determination (R^2^) |
| Nichols et al., 2022 [40] | Accuracy, precision, recall |
| Oladeji et al., 2021 [41] | Root mean square error (RMSE), coefficient of determination (R^2^) |
| Park et al., 2024 [42] | Accuracy, sensitivity |
| Park and Edington, 2004 [43] | Accuracy, AUC ROC, F1-score |
| Park, 2024 [44] | Accuracy, AUC ROC |
| Pereira et al., 2025 [45] | Accuracy, precision, recall, F1-score |
| Puterman et al., 2020 [46] | Not reported |
| Qasrawi et al., 2023 [47] | Accuracy, precision, recall, AUC ROC, F1- score, time |
| Recenti, Ricciardi, Edmunds, Gislason, et al., 2021 [48] | Regression: Mean absolute error (MAE), mean squared error (MSE), root mean squared error (RMSE), coefficient of determination (R2). Classification: Accuracy, specificity, sensitivity |
| Recenti, Ricciardi, Edmunds, Jacob, et al., 2021 [49] | Accuracy, specificity, sensitivity, precision, recall, AUC ROC, F1-score |
| Ren et al., 2025 [50] | Accuracy, specificity, sensitivity, AUC ROC |
| Ruiz et al., 2024 [51] | Not reported |
| Sandri et al., 2025 [52] | Precision, recall, AUC ROC, F1-score |
| Sathyanarayana et al., 2016 [53] | Accuracy, precision, recall, AUC ROC, F1-score |
| Shi et al., 2025 [54] | Accuracy, precision, recall, specificity, AUC ROC, F1-score, CCA, and Decision Curve Analysis (DCA) |
| Staudenmayer et al., 2015 [55] | Regression: Root mean squared error (RMSE). Classification: Accuracy |
| Stemmer et al., 2022[56] | Precision, recall, AUC ROC, F1-score |
| Su et al., 2025[57] | Accuracy, specificity, sensitivity, AUC ROC, F1-score |
| Wallace et al., 2019 [58] | Not reported |
| Wallace et al., 2021 [59] | Not reported |
| Wang et al., 2025 [60] | Accuracy, specificity, sensitivity, AUC ROC, DCA |
| Xin & Ren, 2022 [61] | Accuracy, specificity, sensitivity, AUC ROC |
| Zhang et al., 2024 [62] | Accuracy, specificity, precision, recall, AUC ROC, F1-score |
| Zhou et al., 2019 [63] | AUC ROC |
| Zhou et al., 2022 [64] | Root mean squared error (RMSE), coefficient of determination (R2) |
| Zhou et al., 2025 [65] | Accuracy, specificity, sensitivity, AUC ROC, F1-score |

References

1. Abdul Rahman H, Kwicklis M, Ottom M, Amornsriwatanakul A, H. Abdul-Mumin K, Rosenberg M, Dinov ID. Machine Learning-Based Prediction of Mental Well-Being Using Health Behavior Data from University Students. Bioengineering 2023 May 10;10(5):575. doi: 10.3390/bioengineering10050575

2. Afrash MR, Bayani A, Shanbehzadeh M, Bahadori M, Kazemi-Arpanahi H. Developing the breast cancer risk prediction system using hybrid machine learning algorithms. Journal of education and health promotion 2022;11:272. PMID:36325225

3. Ai M, Morris TP, Zhang J, de la Colina AN, Tremblay-Mercier J, Villeneuve S, Whitfield-Gabrieli S, Kramer AF, Geddes MR, Aisen P, Anthal E, Appleby M, Bellec P, Benbouhoud F, Bohbot V, Brandt J, Breitner JCS, Brunelle C, Chakravarty M, Cheewakriengkrai L, Collins L, Couture D, Craft S, Dadar M, Daoust L-A, Das S, Dauar-Tedeschi M, Dea D, Desrochers N, Dubuc S, Duclair G, Dufour M, Eisenberg M, El-Khoury R, Etienne P, Evans A, Faubert A-M, Ferdinand F, Fonov V, Fontaine D, Francoeur R, Frenette J, Gagné G, Gauthier S, Gervais V, Giles R, Gonneaud J, Gordon R, Greco C, Hoge R, Hudon L, Ituria-Medina Y, Kat J, Kazazian C, Kligman S, Kostopoulos P, Labonté A, Lafaille-Magnan M-E, Lee T, Leoutsakos J-M, Leppert I, Madjar C, Mahar L, Maltais J-R, Mathieu A, Mathotaarachchi S, Mayrand G, McSweeney M, Meyer P-F, Michaud D, Miron J, Morris JC, Multhaup G, Münter L-M, Nair V, Near J, Newbold-Fox H, Nilsson N, Pagé V, Pascoal TA, Petkova M, Picard C, Binette AP, Pogossova G, Poirier J, Rajah N, Remz J, Rioux P, Rosa-Neto P, Sager MA, Saint-Fort EF, Savard M, Soucy J-P, Sperling RA, Spreng N, St-Onge F, Tardif C, Théroux L, Thomas RG, Toussaint P-J, Tuwaig M, Vachon-Presseau E, Vallée I, Venugopalan V, Wan K, Wang S. Resting-state MRI functional connectivity as a neural correlate of multidomain lifestyle adherence in older adults at risk for Alzheimer’s disease. Scientific Reports (Nature Publisher Group) Northeastern University, Department of Psychology, Boston, USA (GRID:grid.261112.7) (ISNI:0000 0001 2173 3359) ; Northeastern University, Department of Physical Therapy, Movement and Rehabilitation Sciences, Boston, USA (GRID:grid.261112.7) (ISNI:0000 000: Nature Publishing Group PP - London; 2023;13(1):7487. doi: https://doi.org/10.1038/s41598-023-32714-1

4. Allen B. An interpretable machine learning model of cross-sectional US county-level obesity prevalence using explainable artificial intelligence. PLOS ONE 2023;18(10). doi: 10.1371/journal.pone.0292341

5. Alshurafa N, Sideris C, Pourhomayoun M, Kalantarian H, Sarrafzadeh M, Eastwood J-A. Remote Health Monitoring Outcome Success Prediction Using Baseline and First Month Intervention Data. IEEE Journal of Biomedical and Health Informatics 2017 Mar;21(2):507–514. PMID:26780823

6. Birk N, Matsuzaki M, Fung TT, Li Y, Batis C, Stampfer MJ, Deitchler M, Willett WC, Fawzi WW, Bromage S, Kinra S, Bhupathiraju SN, Lake E. Exploration of Machine Learning and Statistical Techniques in Development of a Low-Cost Screening Method Featuring the Global Diet Quality Score for Detecting Prediabetes in Rural India. The Journal of Nutrition 2021 Oct;151(12 Suppl 2):110S-118S. PMID:34689190

7. Bôto JM, Marreiros A, Diogo P, Pinto E, Mateus MP, Boto JM, Marreiros A, Diogo P, Pinto E, Mateus MP, Bôto JM, Marreiros A, Diogo P, Pinto E, Mateus MP. Health behaviours as predictors of the Mediterranean diet adherence: a decision tree approach. PUBLIC HEALTH NUTRITION 2022 Aug;25(7):1864–1876. PMID:34369348

8. Butkevičiūtė E, Bikulčienė L, Žvironienė A, Butkeviciute E, Bikulciene L, Zvironiene A, Butkevičiūtė E, Bikulčienė L, Žvironienė A, Butkeviciute E, Bikulciene L, Zvironiene A. Physiological State Evaluation in Working Environment Using Expert System and Random Forest Machine Learning Algorithm. HEALTHCARE 2023 Jan;11(2). PMID:36673588

9. Cai T, Long J, Kuang J, You F, Zou T, Wu L. Applying machine learning methods to develop a successful aging maintenance prediction model based on physical fitness tests. Geriatrics & Gerontology International 2020 June;20(6):637–642. PMID:32358851

10. Cheung YK, Hsueh P-YSYS, Qian M, Yoon S, Meli L, Diaz KM, Schwartz JE, Kronish IM, Davidson KW. Are Nomothetic or Ideographic Approaches Superior in Predicting Daily Exercise Behaviors? Analyzing N-of-1 mHealth Data. METHODS OF INFORMATION IN MEDICINE 2017 Feb;56(06):452–460. PMID:29582914

11. Chiang P-H, Dey S. Offline and Online Learning Techniques for Personalized Blood Pressure Prediction and Health Behavior Recommendations. IEEE Access 2019;7:130854–130864. doi: 10.1109/ACCESS.2019.2939218

12. Cortés-Ibañez FO, Nagaraj SB, Cornelissen L, Navis GJ, van der Vegt B, Sidorenkov G, de Bock GH, Cohort P, Sidorenkov G, Bock GHD. Prediction of Incident Cancers in the Lifelines Population-Based Cohort. Cancers 2021 Apr;13(9):2133. doi: 10.3390/cancers13092133

13. Cortés-Ibañez FO, Nagaraj SB, Cornelissen L, Sidorenkov G, de Bock GH, Bock GHD. A Classification Approach for Cancer Survivors from Those Cancer-Free, Based on Health Behaviors: Analysis of the Lifelines Cohort. CANCERS 2021;13(10). doi: 10.3390/cancers13102335

14. Dianati-Nasab M, Salimifard K, Mohammadi R, Saadatmand S, Fararouei M, Hosseini KS, Jiavid-Sharifi B, Chaussalet T, Dehdar S. Machine learning algorithms to uncover risk factors of breast cancer: insights from a large case-control study. FRONTIERS IN ONCOLOGY 2024;13:1276232. PMID:38425674

15. Faruqui SHA, Du Y, Meka R, Alaeddini A, Li C, Shirinkam S, Wang J. Development of a Deep Learning Model for Dynamic Forecasting of Blood Glucose Level for Type 2 Diabetes Mellitus: Secondary Analysis of a Randomized Controlled Trial. JMIR mHealth and uHealth 2019 Nov;7(11):e14452. PMID:31682586

16. Gu X, Li Q, Wang X. Using Life’s Essential 8 and heavy metal exposure to determine infertility risk in American women: a machine learning prediction model based on the SHAP method. Front Endocrinol (Lausanne) Switzerland; 2025;16:1586828. PMID:40687585

17. Guthrie NL, Carpenter J, Edwards KL, Appelbaum KJ, Dey S, Eisenberg DM, Katz DL, Berman MA. Emergence of digital biomarkers to predict and modify treatment efficacy: machine learning study. BMJ open 2019 July;9(7):e030710. PMID:31337662

18. Hu L, Liu B, Li Y. Ranking sociodemographic, health behavior, prevention, and environmental factors in predicting neighborhood cardiovascular health: A Bayesian machine learning approach. Preventive medicine 2020 Dec;141:106240. PMID:32860821

19. Hu L, Liu B, Ji J, Li Y. Tree-Based Machine Learning to Identify and Understand Major Determinants for Stroke at the Neighborhood Level. Journal of the American Heart Association 2020 Nov;9(22):e016745. PMID:33140687

20. Huang YC, Huang ZS, Yang QR, Jin HJ, Xu TK, Fu YT, Zhu Y, Zhang XY, Chen C. Predicting mild cognitive impairment among Chinese older adults: a longitudinal study based on long short-term memory networks and machine learning. FRONTIERS IN AGING NEUROSCIENCE 2023;15. doi: 10.3389/fnagi.2023.1283243

21. Jin T, Halili A. Predicting the risk of depression in older adults with disability using machine learning: an analysis based on CHARLS data. Front Artif Intell Switzerland; 2025;8:1624171. PMID:40673213

22. Kim J, Jeong K, Lee S, Baek Y. Machine-learning model predicting quality of life using multifaceted lifestyles in middle-aged South Korean adults: a cross-sectional study. BMC public health 2024 Jan;24(1):159. PMID:38212741

23. Kimura N, Aota T, Aso Y, Yabuuchi K, Sasaki K, Masuda T, Eguchi A, Maeda Y, Aoshima K, Matsubara E. Predicting positron emission tomography brain amyloid positivity using interpretable machine learning models with wearable sensor data and lifestyle factors. ALZHEIMERS RESEARCH & THERAPY 2023;15(1). doi: 10.1186/s13195-023-01363-x

24. Kiss O, Alzueta E, Yuksel D, Pohl KM, de Zambotti M, Műller-Oehring EM, Prouty D, Durley I, Pelham WE 3rd, McCabe CJ, Gonzalez MR, Brown SA, Wade NE, Marshall AT, Sowell ER, Breslin FJ, Lisdahl KM, Dick AS, Sheth CS, McCandliss BD, Guillaume M, Van Rinsveld AM, Dowling GJ, Tapert SF, Baker FC. The Pandemic’s Toll on Young Adolescents: Prevention and Intervention Targets to Preserve Their Mental Health. The Journal of adolescent health : official publication of the Society for Adolescent Medicine 2022 Mar;70(3):387–395. PMID:35090817

25. Li Y, Song H. The association between sports social capital and cognitive health: A longitudinal study of middle-aged and elderly adults in China. SSM Popul Health England; 2025 June;30:101778. PMID:40212736

26. Lim J, Kim J, Cheon S. A Deep Neural Network-Based Method for Early Detection of Osteoarthritis Using Statistical Data. International journal of environmental research and public health 2019 Apr;16(7). PMID:30974803

27. Lim J, Jeong CY, Lim JM, Chung S, Kim G, Noh KJ, Jeong H. Assessing Sleep Quality Using Mobile EMAs: Opportunities, Practical Consideration, and Challenges. IEEE Access 2022;10:2063–2076. doi: 10.1109/ACCESS.2021.3140074

28. Lin Y, Li C, Wang X, Li H. Development of a machine learning-based risk assessment model for loneliness among elderly Chinese: a cross-sectional study based on Chinese longitudinal healthy longevity survey. BMC Geriatr England; 2024 Nov 14;24(1):939. PMID:39543473

29. Liu X, Luo Z, Jing F, Ren H, Li C, Wang L, Chen T. Estimating cardiovascular mortality in patients with hypertension using machine learning: The role of depression classification based on lifestyle and physical activity. J Psychosom Res England; 2025 Feb;189:112030. PMID:39752763

30. Luo L, Yuan J, Xu C, Xu H, Tan H, Shi Y, Zhang H, Xi H. Mental Health Issues and 24-Hour Movement Guidelines-Based Intervention Strategies for University Students With High-Risk Social Network Addiction: Cross-Sectional Study Using a Machine Learning Approach. J Med Internet Res Canada; 2025 June 13;27:e72260. PMID:40512996

31. Luo W, Gong L, Chen X, Gao R, Peng B, Wang Y, Luo T, Yang Y, Kang B, Peng C, Ma L, Mei M, Liu Z, Li Q, Yang S, Wang Z, Hu J. Lifestyle and chronic kidney disease: A machine learning modeling study. Frontiers in nutrition 2022;9:918576. PMID:35938107

32. Luo Y, Guo M, Zhang Q. Cross-national analysis of social determinants of frailty among middle-aged and older adults: a machine learning study in the USA, England, and China. HUMANITIES & SOCIAL SCIENCES COMMUNICATIONS 2025 May 30;12(1). doi: 10.1057/s41599-025-05088-0

33. Majcherek D, Kowalski AM, Lewandowska MS. Lifestyle, Demographic and Socio-Economic Determinants of Mental Health Disorders of Employees in the European Countries. International Journal of Environmental Research and Public Health 2022 Sept;19(19):11913. PMID:36231214

34. Majcherek D, Ciesielski A, Sobczak P. AI-driven analysis of diabetes risk determinants in U.S. adults: Exploring disease prevalence and health factors. PLoS One United States; 2025;20(9):e0328655. PMID:40901823

35. Matta SC, Sankari Z, Rihana S. Heart rate variability analysis using neural network models for automatic detection of lifestyle activities. BIOMEDICAL SIGNAL PROCESSING AND CONTROL 2018;42:145–157. doi: 10.1016/j.bspc.2018.01.016

36. Moon Y, Woo H. Key risk factors of generalized anxiety disorder in adolescents: machine learning study. Front Public Health Switzerland; 2024;12:1504739. PMID:39839408

37. Morris MC, Moradi H, Aslani M, Sims M, Schlundt D, Kouros CD, Goodin B, Lim C, Kinney K. Predicting incident cardiovascular disease among African-American adults: A deep learning approach to evaluate social determinants of health in the Jackson heart study. PLOS ONE 2023;18(11). doi: 10.1371/journal.pone.0294050

38. Mousavi H, Karandish M, Jamshidnezhad A, Hadianfard AM. Determining the effective factors in predicting diet adherence using an intelligent model. Scientific reports 2022 July;12(1):12340. PMID:35853992

39. Mun E-Y, Geng F. Predicting post-experiment fatigue among healthy young adults: Random forest regression analysis. Psychological test and assessment modeling 2019 Nov;61(4):471–493. PMID:32038903

40. Nichols ES, Pathak HS, Bgeginski R, Mottola MF, Giroux I, Van Lieshout RJ, Mohsenzadeh Y, Duerden EG. Machine learning-based predictive modeling of resilience to stressors in pregnant women during COVID-19: A prospective cohort study. PloS one 2022;17(8):e0272862. PMID:35951588

41. Oladeji O, Zhang C, Moradi T, Tarapore D, Stokes AC, Marivate V, Sengeh MD, Nsoesie EO. Monitoring Information-Seeking Patterns and Obesity Prevalence in Africa With Internet Search Data: Observational Study. JMIR public health and surveillance 2021 Apr;7(4):e24348. PMID:33913815

42. Park H, Jung SY, Han MK, Jang Y, Moon YR, Kim T, Soo-Yong S, Hwang H. Lowering Barriers to Health Risk Assessments in Promoting Personalized Health Management. Journal of Personalized Medicine KakaoHealthCare Corp., Seongnam-si 13529, Gyeonggi-do, Republic of Korea; hpark.park@kakaohealthcare.com (H.P.); saylor.moon@kakaohealthcare.com (Y.R.M.); ray0601@snu.ac.kr (T.K.); sean.shin@kakaohealthcare.com (S.-Y.S.); drhwang.spike@kakaohealthcare.com: MDPI AG PP - Basel; 2024;14(3):316. doi: https://doi.org/10.3390/jpm14030316

43. Park J, Edington DW. Application of a prediction model for identification of individuals at diabetic risk. Methods of information in medicine 2004;43(3):273–281. PMID:15227557

44. Park S. Association of a High Healthy Eating Index Diet with Long-Term Visceral Fat Loss in a Large Longitudinal Study. Nutrients 2024 Feb;16(4). PMID:38398858

45. Pereira MG, Santos M, Magalhães R, Rodrigues C, Araújo O, Durães D. Burnout Risk Profiles in Psychology Students: An Exploratory Study with Machine Learning. Behav Sci (Basel) Switzerland; 2025 Apr 9;15(4). PMID:40282126

46. Puterman E, Weiss J, Hives BA, Gemmill A, Karasek D, Mendes WB, Rehkopf DH. Predicting mortality from 57 economic, behavioral, social, and psychological factors. Proceedings of the National Academy of Sciences of the United States of America 2020 July;117(28):16273–16282. PMID:32571904

47. Qasrawi R, Polo SV, Abu Khader R, Abu Al-Halawa D, Hallaq S, Abu Halaweh N, Abdeen Z. Machine learning techniques for identifying mental health risk factor associated with schoolchildren cognitive ability living in politically violent environments. FRONTIERS IN PSYCHIATRY 2023;14. doi: 10.3389/fpsyt.2023.1071622

48. Recenti M, Ricciardi C, Edmunds KJ, Gislason MK, Sigurdsson S, Carraro U, Gargiulo P. Healthy Aging Within an Image: Using Muscle Radiodensitometry and Lifestyle Factors to Predict Diabetes and Hypertension. IEEE journal of biomedical and health informatics 2021 June;25(6):2103–2112. PMID:33306475

49. Recenti M, Ricciardi C, Edmunds K, Jacob D, Gambacorta M, Gargiulo P. Testing soft tissue radiodensity parameters interplay with age and self-reported physical activity. European journal of translational myology 2021 July;31(3). PMID:34251162

50. Ren H, Zheng Y, Li C, Jing F, Wang Q, Luo Z, Li D, Liang D, Tang W, Liu L, Cheng W. Using Machine Learning to Predict Cognitive Decline in Older Adults From the Chinese Longitudinal Healthy Longevity Survey: Model Development and Validation Study. JMIR Aging Canada; 2025 Apr 30;8:e67437. PMID:40305830

51. Ruiz F, Bustamante D, Gonzalez K, Sandoval K. Classification Tree for the Identification of Symptoms Associated with Depression in Students of a Public University. RETOS-NUEVAS TENDENCIAS EN EDUCACION FISICA DEPORTE Y RECREACION 2024;(52):104–114.

52. Sandri E, Cerdá Olmedo G, Piredda M, Werner LU, Dentamaro V. Explanatory AI Predicts the Diet Adopted Based on Nutritional and Lifestyle Habits in the Spanish Population. Eur J Investig Health Psychol Educ Switzerland; 2025 Jan 24;15(2). PMID:39997075

53. Sathyanarayana A, Joty S, Fernandez-Luque L, Ofli F, Srivastava J, Elmagarmid A, Arora T, Taheri S. Sleep Quality Prediction From Wearable Data Using Deep Learning. JMIR Mhealth Uhealth 2016 Nov 4;4(4):e125. doi: 10.2196/mhealth.6562

54. Shi H, Fang Y, Ma X. Application of machine learning algorithms in osteoporosis analysis based on cardiovascular health assessed by life’s essential 8: a cross-sectional study. J Health Popul Nutr Bangladesh; 2025 May 29;44(1):180. PMID:40442859

55. Staudenmayer J, He S, Hickey A, Sasaki J, Freedson P. Methods to estimate aspects of physical activity and sedentary behavior from high-frequency wrist accelerometer measurements. Journal of applied physiology (Bethesda, Md : 1985) 2015 Aug;119(4):396–403. PMID:26112238

56. Stemmer M, Parmet Y, Ravid G. Identifying Patients With Inflammatory Bowel Disease on Twitter and Learning From Their Personal Experience: Retrospective Cohort Study. Journal of medical Internet research 2022 Aug;24(8):e29186. PMID:35917151

57. Su W, Jia H, Chang W, Jiang S, Dong S, Ge H, Qi Y, Li X, Ma G. The secrets of medical students’ psychological resilience: a dual perspective of machine learning and path analysis. Int J Med Inform Ireland; 2025 Sept 3;205:106111. PMID:40912161

58. Wallace ML, Buysse DJ, Redline S, Stone KL, Ensrud K, Leng Y, Ancoli-Israel S, Hall MH. Multidimensional Sleep and Mortality in Older Adults: A Machine-Learning Comparison With Other Risk Factors. The journals of gerontology Series A, Biological sciences and medical sciences 2019 Nov;74(12):1903–1909. PMID:30778527

59. Wallace ML, Coleman TS, Mentch LK, Buysse DJ, Graves JL, Hagen EW, Hall MH, Stone KL, Redline S, Peppard PE. Physiological sleep measures predict time to 15-year mortality in community adults: Application of a novel machine learning framework. Journal of sleep research 2021 Dec;30(6):e13386. PMID:33991144

60. Wang X, Zhang D, Lu L, Meng S, Li Y, Zhang R, Zhou J, Yu Q, Zeng L, Zhao J, Zeng Y, Gao R. Development and validation of an explainable machine learning model for predicting the risk of sleep disorders in older adults with multimorbidity: a cross-sectional study. Front Public Health Switzerland; 2025;13:1619406. PMID:40860561

61. Xin Y, Ren X. Predicting depression among rural and urban disabled elderly in China using a random forest classifier. BMC psychiatry 2022 Feb;22(1):118. PMID:35168579

62. Zhang L, Zhao S, Yang Z, Zheng H, Lei M. An Artificial Intelligence Platform to Stratify the Risk of Experiencing Sleep Disturbance in University Students After Analyzing Psychological Health, Lifestyle, and Sports: A Multicenter Externally Validated Study. Psychology research and behavior management 2024;17:1057–1071. PMID:38505352

63. Zhou M, Fukuoka Y, Goldberg K, Vittinghoff E, Aswani A. Applying machine learning to predict future adherence to physical activity programs. BMC medical informatics and decision making 2019 Aug;19(1):169. PMID:31438926

64. Zhou RZ, Hu Y, Tirabassi JN, Ma Y, Xu Z. Deriving neighborhood-level diet and physical activity measurements from anonymized mobile phone location data for enhancing obesity estimation. International journal of health geographics 2022 Dec;21(1):22. PMID:36585658

65. Zhou X, Wang Y, Pan Z, Chang Q, Zhu Y, Zhou G, Zhang G, Zhang Y, Chen X, Liu H. Lifestyle-associated serum metabolites profiling in relation to risk of late-onset psoriasis. J Eur Acad Dermatol Venereol England; 2025 Sept 16; PMID:40956048
