# Supplementary material for: Machine Learning for the Analysis of Healthy Lifestyle Data: Scoping Review and Guidelines
Source: JMIR Hum Factors. 2026 Feb 27;13:e78648. doi: 10.2196/78648 (PMC12954701; doi:10.2196/78648)
Supplement: Checklist 2 [file humanfactors-v13-e78648-s003.docx]

Checklist 2. Reporting checklist for machine learning analysis in healthy lifestyle data.

| **Reporting Checklist for Machine Learning Analysis in Healthy Lifestyle Data** | | |
| --- | --- | --- |
| **Health outcome** |  | |
| **Data Acquisition** | **Data repository** |  |
| Self-acquired |  |  |
| Public dataset |  |  |
| Private dataset |  |  |
| **Acquisition Methods** | **Specification** |  |
| Physical activity |  |  |
| Diet |  |  |
| Sleep |  |  |
| Stress |  |  |
| Other |  |  |
| **Preprocessing & Feature engineering** | **Specification** |  |
| Transformation |  |  |
| Missing imputation |  |  |
| Resampling |  |  |
| Dimensionality reduction |  |  |
| **Supervised Machine Learning** | **Specification** |  |
| Tree-based |  |  |
| Support vector machine |  |  |
| Deep learning |  |  |
| Other |  |  |
| Algorithm hyperparameters |  |  |
| **Model Evaluation** |  |  |
| Train and test |  |  |
| k-fold cross-validation |  |  |
| Leave-one-out |  |  |
| Rolling forecast |  |  |
| Other |  |  |
| **Performance metrics** |  |  |
| Accuracy |  |  |
| Area Under the curve ROC (AUC ROC) |  |  |
| Specificity |  |  |
| Sensitivity |  |  |
| Mean absolute error (MAE) |  |  |
| Root mean square error (RMSE) |  |  |
| Other |  |  |
| **Explainability** |  |  |
| SHAP values |  |  |
| LIME |  |  |
| **Software** | **Code repository** |  |
| R |  |  |
| Python |  |  |
| Other |  |  |
